# A circadian checkpoint relocates neutrophils to minimize injury

**DOI:** 10.1084/jem.20250240

**Published:** 2025-12-12

**Authors:** Alejandra Aroca-Crevillén, Sandra Martín-Salamanca, Lidiane S. Torres, Georgiana Crainiciuc, Jon Sicilia, Eduardo Peñaloza-Martínez, Nicolás Rosillo, Miguel Molina-Moreno, Jose M. Adrover, Andrea Rubio-Ponce, Tommaso Vicanolo, Xiaosong Liu, Kanin Wichapong, Vanessa Núñez, Karl Balabanian, Françoise Bachelerie, David Sancho, María Casanova-Acebes, José T. Ortiz-Pérez, María Á. Moro, Héctor Bueno, Gerry A. F. Nicolaes, Andrés Hidalgo

**Affiliations:** 1 Spanish National Centre for Cardiovascular Research Carlos III, Madrid, Spain; 2 Department of Cell Biology and Ruth L. and David S. Gottesman Institute for Stem Cell Biology and Regenerative Medicine, Albert Einstein College of Medicine, Bronx, New York, NY 10461, USA; 3 Cardiology Department. Hospital Universitario 12 de Octubre, Madrid, Spain; 4 Instituto de Investigación Sanitaria Hospital 12 de Octubre (imas12), Madrid, Spain; 5 Centro de Investigación Biomédica en Red Enfermedades Cardiovasculares (CIBERCV), Madrid, Spain; 6 Vascular Biology and Therapeutics Program and Department of Immunobiology, Yale University School of Medicine, New Haven, USA; 7 Cancer Macroenvironment Lab, The Francis Crick Institute, London, UK; 8 Department of Biochemistry, Cardiovascular Research Institute Maastricht (CARIM), Maastricht University, the Netherlands; 9 Hillmark BV, Oxfordlaan 70, 6229 EV, Maastricht, the Netherlands; 10 Université Paris Cité, Institut de Recherche Saint-Louis, INSERM U1342, Paris, France; 11 Université Paris-Saclay, Inserm, Inflammation, Microbiome and Immunosurveillance, Orsay, France; 12 Cancer Immunity Laboratory, Spanish National Cancer Centre, Madrid, Spain; 13 Institut Clínic Cardiovascular, Hospital Clínic Barcelona and Institut d’investigacions Biomèdiques August Pi i Sunyer (IDIBAPS), University of Barcelona, Barcelona, Spain

**Keywords:** neutrophil, circadian, CXCR4 agonist, vascular damage

## Abstract

Inflammation-driven injury, a significant source of morbidity and mortality worldwide, is largely mediated by the cytotoxic activities of neutrophils, which extend the initial lesion and jeopardize organ function. Intriguingly, inflammatory injury naturally declines at specific times of day, suggesting that circadian mechanisms exist that mitigate the destructive activity of neutrophils and protect the host. Here, we show that the periods of diurnal protection coincide with peaks in plasma CXCL12, a chemokine that inhibits the neutrophil-intrinsic circadian clock by signaling through CXCR4. Genetic deletion of this clock, or a hyperactive form of CXCR4, prevented the diurnal spikes of injury, and treatment with a synthetic CXCR4 agonist conferred protection from myocardial and vascular injury. In tissues, this protection was mediated by repositioning neutrophils in the wound core, which spared neighboring host cells from apoptotic death. Thus, a circadian neutrophil checkpoint protects from exuberant inflammation and can be activated to protect the host.

## Introduction

Neutrophils are the first responders to trauma and infections (Aroca-Crevillén et al., 2024; Mayadas et al., 2014). While essential to prevent microbial invasion, their highly cytotoxic activity can elicit irreversible damage to bystander host cells. This is relevant both in the context of infections and sterile inflammation, conditions in which the affected tissue recruits large numbers of neutrophils that can expand the area of damage by releasing an arsenal of chemicals and cellular components, and induce the death of unaffected neighboring cells (Aroca-Crevillén et al., 2024; Silvestre-Roig et al., 2020; Wang et al., 2017). These antagonistic effects of immune protection and inflammatory injury have precluded the development of effective therapies because both properties are generally considered inseparable features of neutrophils. Recent studies, however, have demonstrated that neutrophils are not homogeneous across tissues, disease states, or diurnal time (Adrover et al., 2019; Casanova-Acebes et al., 2013; Ng et al., 2019; Wigerblad et al., 2022; Xie et al., 2020; Zhang et al., 2015), raising the possibility of targeting neutrophils in a spatial and temporal-specific manner, as shown for monocytes in the context of atherosclerosis (Huo et al., 2017; Winter et al., 2018). The temporal regulation of innate immune responses is of particular interest given the stark variations in the response to infection seen at different times of day and the well-known circadian variations in the onset and magnitude of inflammatory responses (Bellet et al., 2013; Edgar et al., 2016; Gibbs et al., 2014; Hand et al., 2016). This is relevant for cardiovascular diseases (CVD), in which both experimental and clinical studies have shown that ischemic events in the early morning have more severe consequences in myocardial death and future cardiac performance (Adrover et al., 2019; Muller et al., 1985; Suárez-Barrientos et al., 2011; Reiter et al., 2012), and correlate with the number of neutrophils in the circulation.

While studying the diurnal regulation of inflammation, we previously identified a cell-intrinsic circadian clock that controlled the transcription, granule content, and migratory properties of neutrophils in the circulation, a phenomenon collectively referred to as neutrophil *aging* (Adrover et al., 2020, 2019; Casanova-Acebes et al., 2013). This neutrophil clock is controlled by the transcription factor Bmal1, which regulates the expression of CXCL2 to activate neutrophils in a cell-autonomous manner through CXCR2. Consequently, mice with neutrophil-specific ablation of Bmal1 (referred to as Bmal1^ΔN^ mice) showed arrhythmic responses to infections (Adrover et al., 2019). Notably, these studies also identified the chemokine receptor CXCR4 as a negative regulator of this clock, such that mice in which neutrophils specifically lacked the receptor (CXCR4^ΔN^ mice) featured constitutive circadian aging and mounted stronger anti-microbial responses, but succumbed to experimental sterile injury (Adrover et al., 2019). Intriguingly, inhibition of circadian aging in Bmal1^ΔN^ mice did not impair the response to bacterial or fungal infections (Adrover et al., 2019), revealing a dissociation in the control of the inflammatory and antimicrobial activities of neutrophils.

Here, we posited that targeting the neutrophil clock could provide a simple and effective means to blunt the toxic activity of these cells during cardiovascular inflammation without compromising antimicrobial defense. To gain mechanistic and functional insights, we used a variety of complementary models of inflammation across different tissues (heart, skin, and microvasculature). We show that the circadian spikes in myocardial injury in mice and humans are mediated by neutrophils and controlled by their intrinsic clock. Pharmacological activation of CXCR4 through a synthetic agonist blunted the inflammatory response and protected from vascular occlusion in sickle cell disease and from myocardial infarction but did not compromise the antimicrobial response. Unexpectedly, mechanistic studies in the inflamed skin revealed that the protective effects in tissues were mediated by repositioning neutrophils away from the unaffected tissue, thereby unveiling a circadian checkpoint mediated by CXCR4 activation that protects tissues from the toxic activity of neutrophils.

## Results

### The neutrophil clock controls diurnal variations of ischemic injury

**Figure 1:**
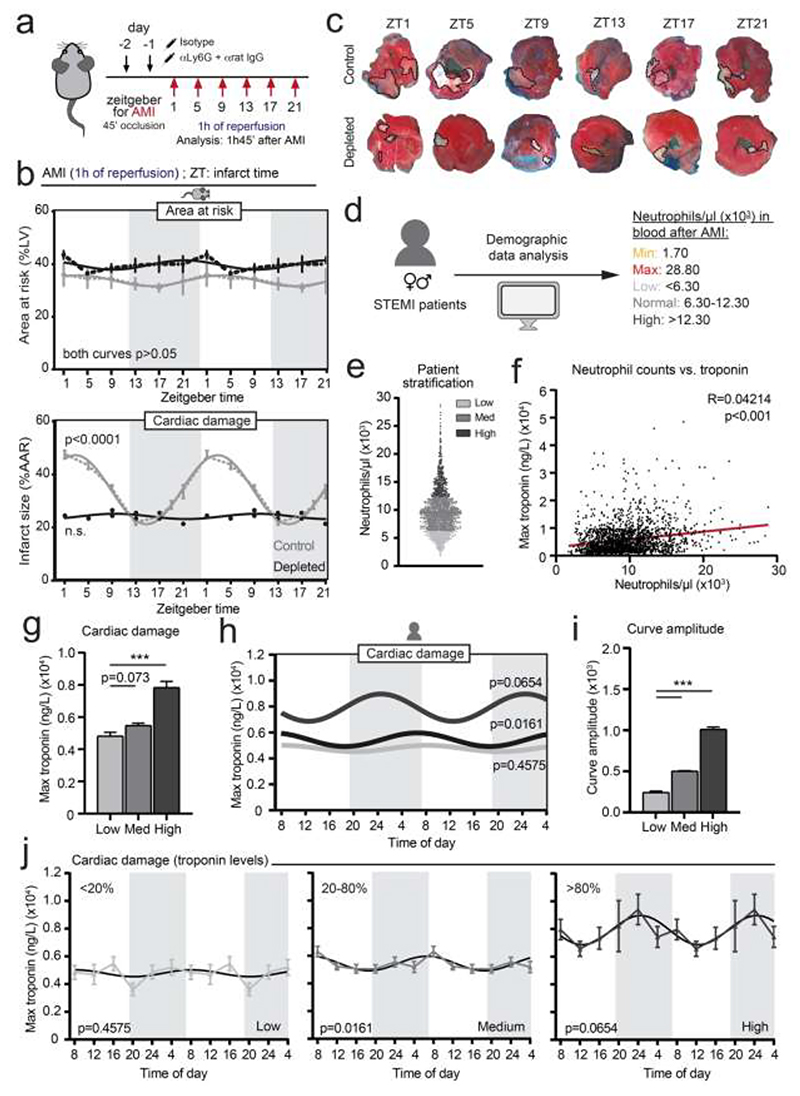
Neutrophil numbers correlate with circadian oscillations in infarct severity in human patients. (a) Experimental scheme for ischemia-reperfusion protocol (AMI model) during a 24-hour cycle and antibody cocktails used as control and to deplete neutrophils. ZT indicates the time at which the infarct was induced. Analyses were performed 1h and 45min later. (b) Areas at risk of the myocardium (top) in control and neutropenic mice at different circadian times and infarct sizes (down) after correction for areas at risk in the same animals subjected to MI followed by 1h of reperfusion; n=1-2 mice per time point. The indicated p values were calculated after COSINOR adjustment as indicated in the Methods section (amplitude vs. zero test). The diurnal curves are repeated to better appreciate the pattern. (c) Representative images of infarcted hearts from control and neutropenic mice infarcted at the indicated ZT. Dotted black lines highlight areas of dead myocardium. d) Summary of the human cohort of STEMI patients. (e) Distribution of blood neutrophils in the 2041 STEMI patients. (f) Correlation of maximum troponin levels with neutrophils in circulation at admission time. (g) Severity of cardiac injury in the three groups of STEMI patients. (h) Cardiac damage shown as the maximum troponin levels of the cohort of STEMI patients entering the intensive care unit at different times of the day; n=2026 patients. (i) Circadian amplitude of cardiac damage in the three virtual experimental groups of STEMI patients. (j) Cardiac damage shown as the maximum troponin levels for each patient group, depending on the time of admission at the intensive care unit; n=401 (left), 1228 (middle) and 39*5* (right). The diurnal curves are repeated to better appreciate the pattern. Data in (a-c) are from a single experiment. Data are shown as mean ± SEM. *p<0.05; **p<0.01; ***p<0.001, as determined by correlation analysis (f), one-way ANOVA (g, i) and amplitude vs. zero test (b, h and j).

Myocardial infarction, a leading cause of morbidity and death worldwide, represents a paradigm of collateral tissue injury inflicted by neutrophils and follows marked circadian patterns in severity, both in mice (Adrover et al., 2019; Eckle et al., 2012; Schloss et al., 2016; Schofield et al., 2013; Weng et al., 2021) and humans (Suárez-Barrientos et al., 2011; Muller et al., 1985). Because different times of peak damage have been reported depending on the experimental model of ischemic insult (permanent ischemia leading to hypoxic death (Schloss et al., 2016) versus ischemia-reperfusion (Adrover et al., 2019; Eckle et al., 2012; Weng et al., 2021)), we focused here on the ischemia-reperfusion model to explore the contribution of neutrophils to the circadian patterns of injury. We transiently ligated the left anterior descending coronary artery of mice (Adrover et al., 2019; García-Prieto et al., 2017) every four hours for a full diurnal cycle, to induce 45 min of ischemia at the indicated zeitgeber (ZT, time after the onset of light) and analyzed the infarcted left ventricles after 1h of reperfusion (Fig. 1a). In line with previous reports (Adrover et al., 2019; Eckle et al., 2012; Weng et al., 2021), we confirmed marked diurnal variations in cardiac damage in mice, with peaks of myocardial death when infarction was performed at ZT1-5 (8am-12pm in our facility) and a trough at ZT13-17 (nighttime) (Fig. 1a-c), which could not be explained by differences in the area at risk (AAR), a parameter that controls for the effects of surgery (Fig. 1b). Depletion of neutrophils before infarction (with an anti-Ly6G antibody, as in (Boivin et al., 2020)) (Fig. S1a, b) reduced infarct sizes and, more importantly, led to complete loss of the circadian pattern of myocardial injury (Fig. 1a-c). We corroborated these findings in an independent experiment in which control and neutropenic mice were examined only after performing infarction at the peak and trough times (ZT5 and ZT13; Fig. S1c, d), altogether confirming that neutrophils are responsible for the diurnal spikes in ischemic injury. Of note, our neutrophil-depletion strategy (anti-Ly6G antibody treatment) did not interfere with the recruitment of other immune cells to the infarcted myocardium, confirming that the effect was caused by the depletion of neutrophils (Fig.S1e-g).

Myocardial ischemia in humans has been shown to display diurnal variations in incidence and severity (Muller et al., 1985; Suárez-Barrientos et al., 2011). To assess whether these diurnal variations in human myocardial injury were also associated with neutrophils, we performed an *in silico* retrospective analysis using a dataset of 2,043 STEMI (ST-segment elevation myocardial infarction) patients with recorded times of admission, blood counts, and troponin levels in plasma (a measure of myocardial injury ((Babuin and Jaffe, 2005); Fig. 1d and Table 1). We found that neutrophil counts in the circulation at the time of admission correlated positively with the severity of cardiac injury, as scored by the maximum values of cardiac troponin in plasma (Fig. 1f).

We then created virtual groups by stratifying the patients into percentiles of neutrophil numbers (high for patients in the top percentile >80%, intermediate for 20-80%, and low for the lowest 20% patients; Fig. 1e) and asked whether the diurnal variations were blunted when neutrophil counts were naturally low. Troponin levels gradually increased from the low to the medium and high-count groups, as expected (Arruda-Olson et al., 2009; Dogan et al., 2009) (Fig. 1g). Interestingly, the amplitude of circadian variations in disease severity (assessed by troponin levels in plasma) progressively decreased for each group; there were strong oscillations (highest amplitude) in the group with high levels of neutrophils, mild oscillations in the intermediate group, and no oscillations in the low count group (Fig. 1h-j). These analyses also revealed an early morning peak of injury for the cohort with intermediate levels of neutrophils, consistent with previous studies (Muller et., 1985), and an earlier peak for neutrophil-rich patients that is more consistent with the reported circadian patterns of neutrophils in humans (Adrover et al., 2019). These observations support the contention that neutrophils underlie the circadian patterns of myocardial damage in mice and humans.

**Figure 2:**
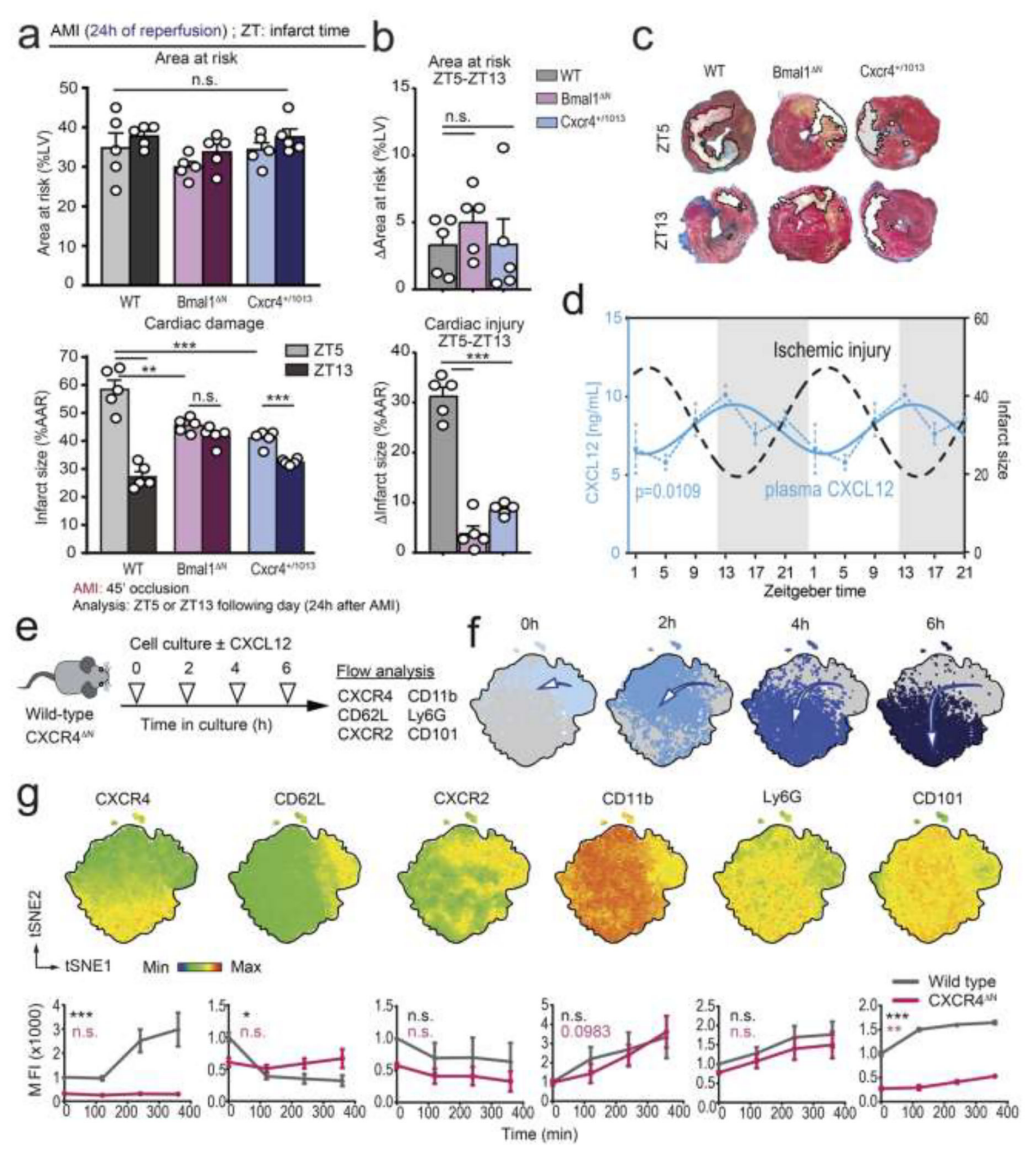
The neutrophil clock controls diurnal oscillations of ischemic injury. (a) Areas at risk in wild-type and mutant mice at ZT5 or ZT13 (up) and infarct sizes after correction for areas at risk (bottom) in the same animals subjected to MI followed by 24h of reperfusion; n=5 mice per group. ZT indicates the time of infarct. Analysis was performed 24h later. (b) Differential area at risk (left) or infarct size (right) between ZT5 and ZT13 in wild-type and mutant mice; n=5 mice per group. (c) Representative images of infarcted hearts from wild-type or mutant mice infarcted at the indicated ZT. Dotted black lines highlight areas of dead myocardium. (d) Diurnal changes of CXCL12 in the plasma of wild-type mice; n=4–9 mice per time point. The diurnal curves are repeated to appreciate the circadian pattern better. (e) Experimental scheme to evaluate the expression of different neutrophil surface markers at different time points after *in vitro* incubation of blood neutrophils from WT and CXCR4^ΔN^ mice. (f) Temporal transition in phenotype (shown as arrows) as determined by surface expression of CXCR4, CD62L, CXCR2, CD11b, Ly6G and CD101 from wild-type blood neutrophils. (g) Kinetics of surface marker expression of blood neutrophils from control (t-distributed stochastic neighbour embedding (tSNE) plots and XY graphs) and CXCR4^ΔN^ (XY graphs) blood neutrophils at 0, 2, 4 and 6h of *in vitro* incubation. n=4-6 mice per group and time point. Data in (a-c) are from a single experiment. Data in (d and e-g) are pooled from two experiments. Data are shown as mean ± SEM. *p<0.05; **p<0.01; ***p<0.001; ns, not significant, as determined by one-way ANOVA (a, b), unpaired t test analysis (a), amplitude vs. zero test (d) and two-way ANOVA (mixed model) (g).

Although the data indicated that neutrophils are responsible for the circadian variations in cardiac inflammatory injury after AMI, they could not discriminate between the effect of neutrophil numbers and their basal activation state at different times of day, a parameter governed by the circadian clock (Adrover et al., 2019). To discern between these possibilities, we induced ischemic injury at ZT5 and ZT13 in Bmal1^ΔN^ mice, in which the circadian clock of neutrophils is genetically disabled (Adrover et al., 2019), and analyzed damage after 24h of reperfusion. Although these mice showed preserved circadian oscillations in neutrophil counts and other hematological parameters in blood (Fig. S2a, b; (Adrover et al., 2019)), they were protected from myocardial tissue death when compared with littermate controls (Fig. 2a, c), as reported (Adrover et al., 2019), despite a moderate but significant increase in neutrophil counts in the circulation of Bmal1^ΔN^ mice (Fig. S2a, b). Importantly, Bmal1^ΔN^ mice showed a complete loss in the diurnal spikes in myocardial injury (Fig. 2a-c), and this was not caused by alterations in organismal circadian activity, as these were preserved in Bmal1^ΔN^ or neutropenic mice placed in metabolic cages (Fig. S2c). We confirmed the protection from cardiac injury at ZT13 and neutropenic mice (at ZT5) by magnetic resonance imaging (MRI) analysis, as well as loss of diurnal oscillations in myocardial injury in the Bmal1^ΔN^ mutants (Fig. S2d-f). Together, these data support the notion that diurnal variations in neutrophil basal activity controlled by the neutrophil circadian clock are responsible for the circadian oscillations in inflammatory injury, raising the possibility of pharmacological targeting of the neutrophil circadian clock to protect from pathogenic inflammation.

### CXCR4 signaling inhibits neutrophil circadian aging and protects from inflammatory injury

CXCR4 is a key regulator of neutrophil trafficking, as shown by premature neutrophil mobilization from the bone marrow (BM) and altered distribution in perivascular niches in the lungs of mice with neutrophil-specific deficiency in CXCR4 (CXCR4^ΔN^ mice) (Ballesteros et al., 2020; Eash et al., 2009; Filippo and Rankin, 2018). Importantly, CXCR4 signaling has also been shown to cross-inhibit CXCR2 (Eash et al., 2010; Martin et al., 2003) and CXCR4^ΔN^ mice display constitutive neutrophil aging, suggesting that CXCR4 acts as a cell-intrinsic inhibitor of the circadian clock in these cells (Adrover et al., 2019). Therefore, we focused on CXCR4 and its natural agonist, CXCL12, in the context of acute myocardial ischemia.

Analysis of the diurnal levels of CXCL12 in the plasma of naïve mice revealed oscillatory levels that were in antiphase with the oscillations in the size of myocardial injury (Fig. 2d), suggesting that diurnal signaling through CXCR4 in neutrophils may confer protection from inflammatory injury. To further define if CXCR4 was a regulator of circadian aging of neutrophils, we devised a simple *in vitro* approach in which we placed blood neutrophils in culture media and analyzed their spontaneous phenotypic change over time by flow cytometry, using a collection of markers previously associated with circadian aging or with maturation, including CXCR4, CD62L, CXCR2, CD11b, Ly6G and CD101 (Adrover et al., 2019; Casanova-Acebes et al., 2013; Evrard et al., 2018; Mackey et al., 2021) (Fig. 2e). We found that, even in these *ex vivo* conditions, neutrophils underwent clear phenotypic transitions over time (Fig. 2f), with changes similar to those reported during *in vivo* aging, including loss of CD62L, and gain of CD11b, Ly6G, CD101 and CXCR4 expression (Fig. 2f, g and (Adrover et al., 2019; Casanova-Acebes et al., 2013)). These temporal changes were absent when we cultured blood neutrophils from CXCR4^ΔN^ mice. Further, these cells showed reduced levels of CD62L, CXCR2 and CD101 that were consistent with an “aged” phenotype (Fig. 2g). Finally, using mice with gain-of-function in CXCR4 signaling due to a point mutation that prevents receptor desensitization (*Cxcr4*^+/1013^ mice, mimicking WHIM syndrome; (Balabanian et al., 2012)), we found these hyperactive CXCR4 mutants were protected from myocardial injury and, notably, showed blunted circadian variations in myocardial injury during AMI (Fig. 2a-c), despite normal organismal circadian activity (Fig. S2a,c). Collectively, these findings indicated that inhibition of the neutrophil clock through activation of CXCR4 prevented the diurnal spikes in neutrophil activation (i.e., circadian aging), suggesting that neutrophil stimulation through this receptor might protect against neutrophil-driven inflammatory injury.

### CXCR4 agonism induces night-like non-pathogenic behaviors in intravascular neutrophils

**Figure 3:**
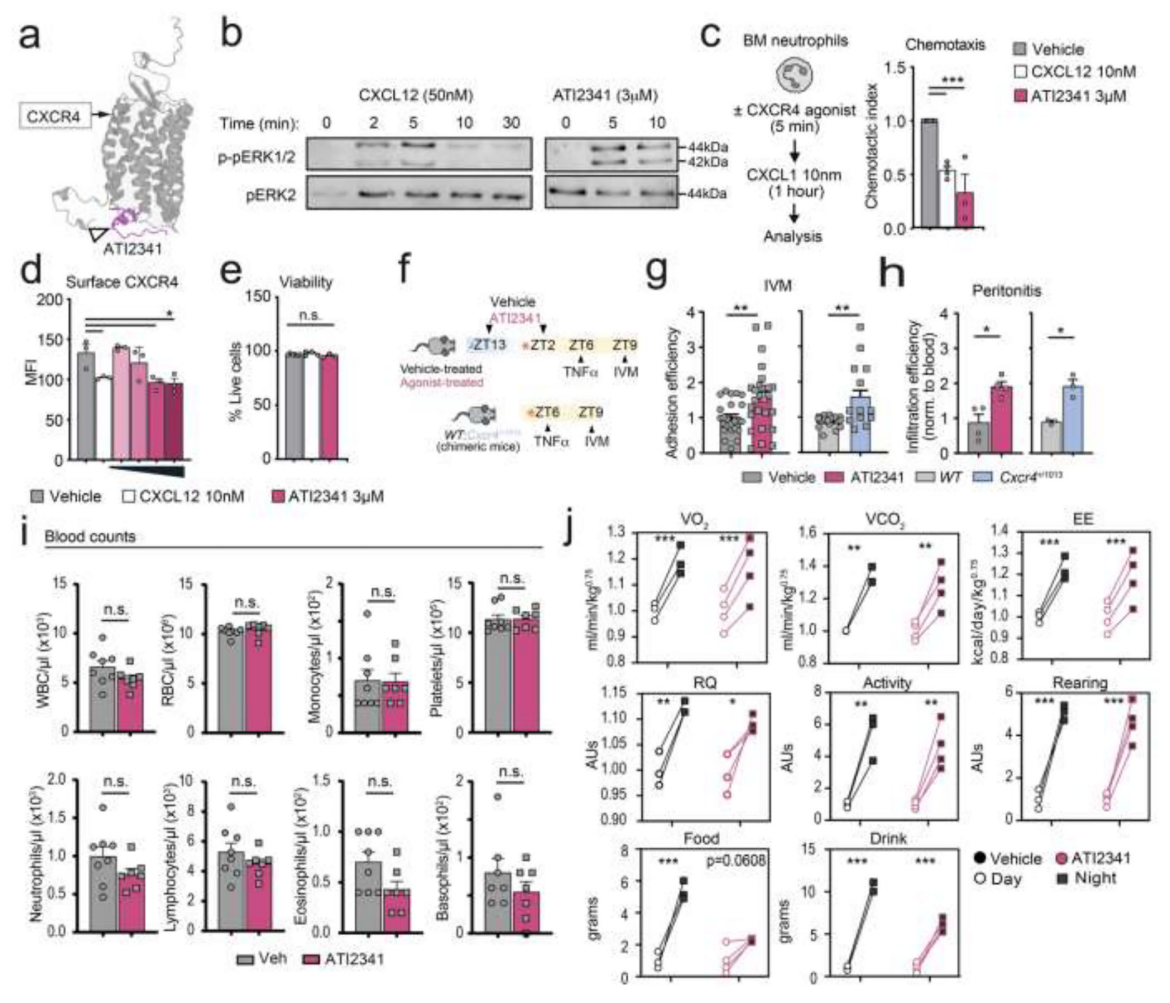
Characterization of ATI2341 *in vitro* and *in vivo*. (a) 3D structure of the CXCR4 agonist pepducin ATI-2341 coupled to CXCR4. (b) p-pERK1/2 protein levels in wild-type BM neutrophils after stimulation with CXCR4 agonists. Blots are representative of n=3 (CXCL12) and 2 (ATI2341) experiments. (c) Inhibition of CXCR2 signaling through CXCL12/CXCR4. Wild-type neutrophils were preincubated with CXCR4 agonists and then allowed to migrate towards a CXCL1 gradient. The number of migrated cells was evaluated by flow cytometry; n=3-6 mice per group. (d) Surface expression of CXCR4 in wild-type neutrophils after stimulation with CXCL12 or dose-dependent concentrations of ATI2341 (0.03, 0.3, 3 and 30µM); n=3 mice per group. (e) Viability of migrated neutrophils evaluated by flow cytometry from mice in (c). (f) Experimental scheme to analyse neutrophil recruitment by IVM during TNFα-induced inflammation. (g) Adhesion of neutrophils on cremasteric venules after treatment with TNFα (inflammation) in vehicle- and ATI2341-treated neutrophils (left) and WT and Cxcr4^+/1013^ (WHIM) neutrophils (right, from transplant chimeras of wild-type and Cxcr4^+/1013^ mutant cells); n=3-4 mice per group, normalized to vehicle or % of blood neutrophils in chimeric mice. (h) Infiltration efficiency of vehicle- and ATI2341-treated neutrophils (left) and WT and Cxcr4^+/1013^ (WHIM) neutrophils into the inflamed peritoneum after zymosan injection; n=3-4 mice per group. (i) Peripheral blood counts in vehicle- and ATI2341-treated mice at ZT5; n=7-8 mice per group. (j) O_2_ consumption (VO2), CO_2_ production (VCO2), energy expenditure (EE), respiratory quotient (RQ), general locomotor activity (activity), vertical activity (rearing), food intake (food) and drink intake (drink) of vehicle- and ATI2341-treated mice housed in metabolic cages for 3 days with food and water available *ad libitum*; n=3-4 mice per group. AUs: Arbitrary Units. Data in (c, e and i) are pooled from two experiments. Data in (d and g, h; right (Cxcr4^+/1013^) are from single experiments. Data in (g, h; left (ATI) and j) are representative of two experiments. Data are shown as mean ± SEM. *p<0.05; **p<0.01; ***p<0.001; ns, not significant, as determined by one-way ANOVA (c, d, e), unpaired t-test (i and g, h; left), paired t-test (g, h; right) and two-way ANOVA (j).

To activate CXCR4 pharmacologically, we selected the commercial compound ATI2341, a 16-amino acid-long pepducin with agonistic effect on this receptor (Fig. 3a and (Tchernychev et al., 2010)). ATI2341 has been shown to induce CXCR4-dependent signaling, receptor internalization, and chemotaxis (Tchernychev et al., 2010). We further confirmed that this compound elicits activation of the ERK1/2 cascade, a central signaling pathway during neutrophil chemotaxis (Filippo and Rankin, 2018; Shi et al., 2020; van der Vorst et al., 2015), with kinetics similar to those of CXCL12 (Fig. 3b). ATI2341 also inhibited the response of neutrophils to CXCR2 ligands (Fig. 3c), and reduced surface CXCR4 to a similar extent as CXCL12, without affecting neutrophil viability (Fig. 3d-e). To examine if activation of CXCR4 with ATI2341 was able to inhibit neutrophil aging (driven by the circadian clock), we next assessed the capacity of the agonist to increase the adhesion and migration of neutrophils *in vivo*, both of which are hallmarks of impaired circadian aging (Adrover et al., 2019). To this end, we administered mice with two doses of the agonist (1mg/kg intraperitoneal, at ZT13 of day -1 and ZT2 of the day of injury; Fig. 3f). This treatment increased the adhesion efficiencies of neutrophils to inflamed vessels (Fig. 3g) as well as their migration in a model of zymosan-induced peritonitis (Fig. 3h), but importantly did not alter the number of neutrophils in blood under homeostatic conditions (probably because it acts acutely in the periphery, compared with WHIM patients, in which persistent hypersignaling in the marrow causes neutropenia (Fig. S2b); (Balabanian et al., 2012)) or organismal circadian activity (Fig. 3i, j). We could reproduce these findings in *Cxcr4*^+/1013^ mice (Fig. 3g-h). Thus, ATI2341 is an efficient agonist of CXCR4 that cross-inhibits CXCR2 signaling in neutrophils and interferes with circadian aging *in vivo*.

**Figure 4:**
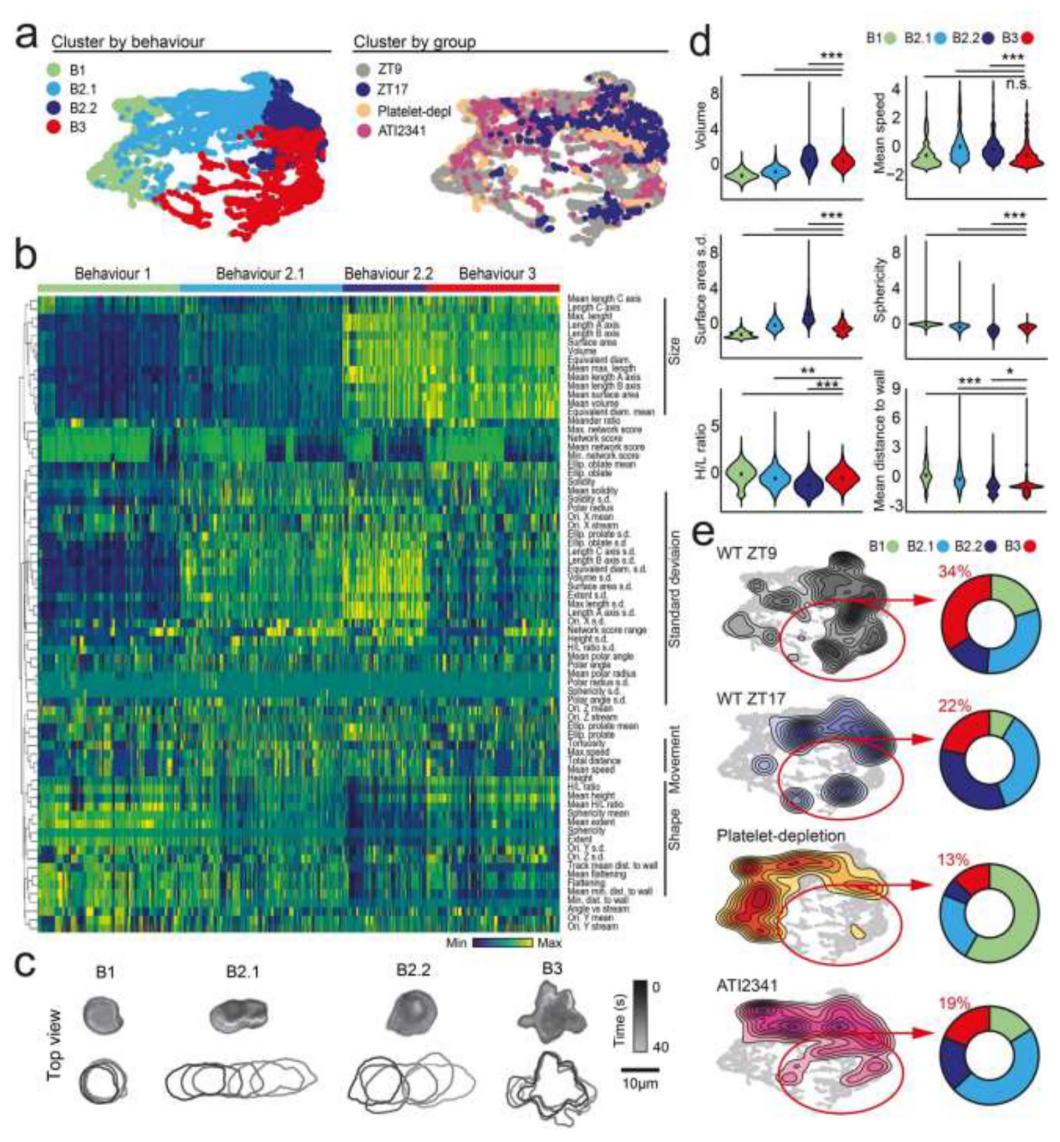
CXCR4 activation reprograms the behavior of intravascular neutrophils. **(a)** Uniform manifold approximation and projection (UMAP) plots showing the four distinct behavioral clusters identified by 4D IVM (left) labelled with colours and distribution of neutrophils from wild-type ZT9 and ZT17, platelet-depleted and ATI-treated mice (right). **(b)** Behavioural profiling: heatmap of the 73 morpho-kinetic parameters obtained for neutrophils inside inflamed venules and distributed for the four identified behavioral profiles. **(c)** Volumetric reconstruction of representative cells from behavioral clusters (top) and temporal outlines (bottom). **(d)** Violin plots for the indicated parameters across the four behavioral groups. **(e)** UMAP (left) and doughnut plots (right) showing the distributions of neutrophils in each experimental group for each behavioral cluster. Numbers are the frequency of intravascular neutrophils with a B3 pathogenic profile. Data in (a-e) are pooled from cells analysed from two experiments. Data are presented as mean ± SEM. *p<0.05; **p<0.01; ***p<0.001; ns, not significant, as determined by one-way ANOVA in (d).

To assess the impact of clock inhibition of neutrophils *in vivo*, we used a recently developed 4D imaging-based platform to examine the presence of pathogenic intravascular neutrophils *in vivo* (Crainiciuc et al., 2022). We used this approach in TNFα-treated mice to obtain 73 morpho-kinetic parameters per cell from hundreds of cells and generated a “behavioral” profile of neutrophils inside inflamed vessels at different circadian times or after ATI2341 treatment. Platelet-depleted mice were used as a reference for neutrophils displaying non-pathogenic intravascular behaviors, as this treatment protects from neutrophil-driven injury (Crainiciuc et al., 2022; Hidalgo et al., 2009; Sreeramkumar et al., 2014). In line with previous studies, we found that intravascular neutrophils displayed different types of behaviors inside inflamed vessels, which we categorized into four different groups (B1, B2.1/B2.2, and B3) (Fig. 4a). These behaviors matched those associated previously with pathogenic and non-pathogenic inflammation (Crainiciuc et al., 2022). For example, B1 featured small spherical cells whose center mass was away from the vessel wall, had reduced motility (Fig. 4b-d), and were enriched in platelet-depleted mice (Fig. 4e). In contrast, B3 identified large neutrophils that flattened against the vessel wall (measured as height/length H/L ratios and distance to the wall; Fig. 4b-d) and were most abundant at daytime (ZT9; Fig. 4e), coinciding with the peak inflammation (Fig. 1b). This B3 behavior has been associated with infarct severity, as inhibiting the Fgr kinase driving this behavior alleviates myocardial injury (Crainiciuc et al., 2022). Finally, B2.1 and B2.2 shared features of high motility and rapid change in shape (measured by high standard deviation values) but differed in size (Fig. 4b-d), likely representing transition states between B1 and B3. Consistent with our hypothesis, we found that mice injured at night-time (ZT17) or treated with ATI2341 featured neutrophils that moved away from the pathogenic B3, into the non-pathogenic B2 behaviors (Fig. 4e). Of note, this dose of ATI2341 did not affect the expression of adhesion molecules on endothelial cells, supporting the contention that the effects were specific to neutrophils. Altogether, these findings supported the contention that inhibition of the neutrophil clock via CXCR4 agonism protects from vascular inflammation by inducing the natural “de-activation” state seen at night, when the neutrophil clock is inactive (Adrover et al., 2019).

### CXCR4 agonism protects from vascular inflammation in sickle cell disease

**Figure 5:**
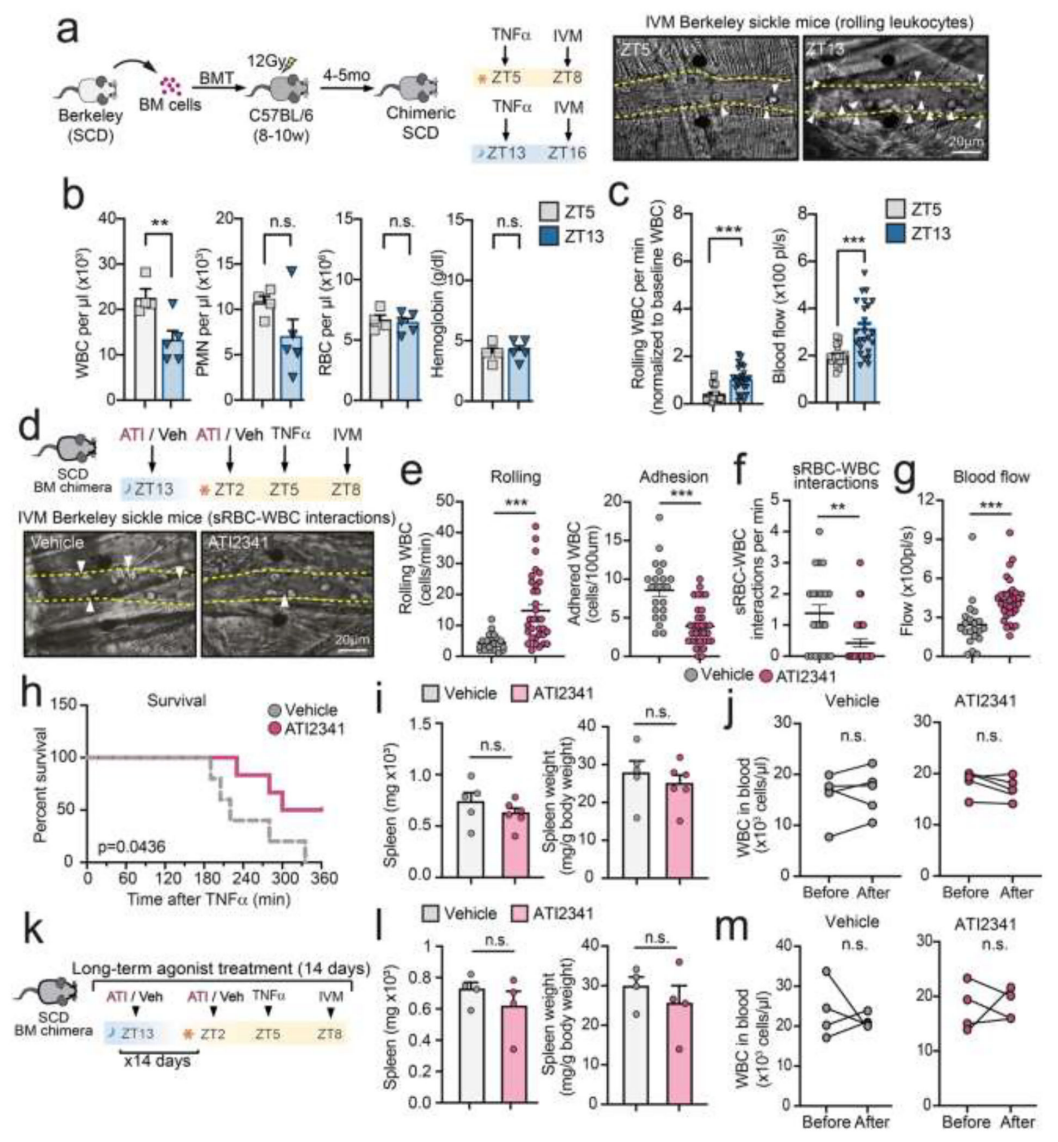
CXCR4 activation protects sickle mice from vascular occlusion. (a) Left, experimental scheme; chimeric SCD mice were generated by transplantation of bone marrow cells (BMT) from transgenic SCD mice into lethally irradiated C57BL/6 male mice. After BM repopulation, TNFα (0.5 μg, i.p.) was administered before intravital imaging of the cremasteric microcirculation at the indicated times. Right, representative intravital microscopy images obtained at different circadian times. Arrows indicate rolling leukocytes. (b) Peripheral blood counts of ZT5 and ZT13 SCD mice collected before TNF-α treatment for baseline reference; n=4-5 mice per group. (c) Number of rolling leukocytes and blood flow rate of ZT5 (daytime) and ZT13 (nighttime) SCD mice after TNFα treatment; n= 4-5 mice per group (2-10 postcapillary venules (15-25μm of diameter) per animal). Rolling values were normalized to the baseline WBC counts. (d) Top, experimental scheme; chimeric SCD mice received two doses of ATI2341 (1 mg/kg, i.p.) or saline control. TNFα (0.5 μg, i.p.) was administered before intravital imaging of the cremasteric microcirculation at the indicated time. Bottom, representative intravital microscopy images. Arrows indicate leukocytes interacting with sRBC. (e) Number of rolling (left) and adhered (right) leukocytes on the endothelium of control and ATI2341-treated SCD mice after TNFα treatment; n=4-5 mice per group (3-7 postcapillary venules per animal). (f, g) Frequency of interactions (f) between sRBCs and leukocytes and blood flow rates (g) from the mice in (e). (h) Survival curves after TNFα treatment in control and ATI2341-treated SCD mice; n=5-6 mice per group. (i) Spleen weights of control and ATI2341-treated SCD mice after TNFα treatment; n=5-6 mice per group. (j) Number of leukocytes in blood of control and ATI2341-treated SCD mice before and after TNFα treatment; n=5 mice per group. (k) Experimental scheme for treatment of chimeric SCD mice with two doses of ATI2341 (1 mg/kg, i.p.) or saline as control for 14 days. TNFα (0.5 μg, i.p.) was administered to induce acute vaso-occlusion and the microcirculation of the cremaster muscle was visualized by intravital microscopy at the indicated time. (l) Spleen weights of control and ATI2341-treated SCD mice (14 days treatment) after TNFα treatment; n=4 mice per group. (m) Number of leukocytes in blood of control and ATI2341-treated SCD mice (14 days treatment) before and after TNFα treatment; n=4 mice per group. Data in (a-m) are from single experiments. Except for survival curves, data are shown as mean ± SEM. **p<0.01; ***p<0.001; ns, not significant, as determined by unpaired t-test analysis (b, c, e, f, g, i, and l), paired t-test analysis (j, m) and long-rank test (h).

To test whether CXCR4 agonism reduced the pathogenic activity of neutrophils in the context of vascular inflammation, we used a model of vaso-occlusion associated with sickle cell disease (SCD), a prototypical model of vascular inflammation driven by hemolytic and rigid sickle erythrocytes (sRBC). This disease is caused by a point mutation in β hemoglobin, leading to endothelial activation and occlusion of vessels through interactions of sRBC with activated neutrophils (Torres and Hidalgo, 2023; Turhan et al., 2002). We used a humanized murine model of SCD (Berkeley model) generated by bone marrow transplantation and subjected to TNFα treatment, which triggers acute vascular inflammation (Turhan et al., 2002), and performed intravital imaging of inflamed cremasteric venules (Fig. 5a). We scored neutrophil recruitment, interactions between sRBC and adherent neutrophils, blood flow as a measure of partial or total occlusion, and death (as in (Turhan et al., 2002)). We first confirmed that SCD mice featured circadian variations in the number of circulating neutrophils, rolling frequency, and blood flow. We found better perfusion at night (Fig. 5a-c), suggesting that vascular occlusion in SCD mice might be alleviated via CXCR4 agonism. Hence, we evaluated whether CXCR4 activation with ATI2341 conferred protection from vascular occlusion and death of the sickle mice (Fig. 5d). ATI2341 treatment induced a marked increase in neutrophil rolling and reduced adhesion (Fig. 5e), suggesting reduced activation of circulating neutrophils. Consistently, treatment with the agonist reduced the capture of sRBC by adherent neutrophils, improved blood flow in the microcirculation, and extended the survival of the sickle mice (Fig. 5f-h). These protective effects occurred despite unaffected blood counts and splenomegaly after ATI2341 treatment (Fig. 5i, j). We found the same protective effect but unaffected splenomegaly after prolonged treatment of sickle mice (for two weeks; Fig. 5k-m), suggesting that the protective effects were caused by a transient and local impact on inflammatory neutrophils rather than by global effects, such as changes in granulopoiesis or sRBC retention in the spleen.

### CXCR4 agonism protects the heart from ischemia/reperfusion injury

**Figure 6:**
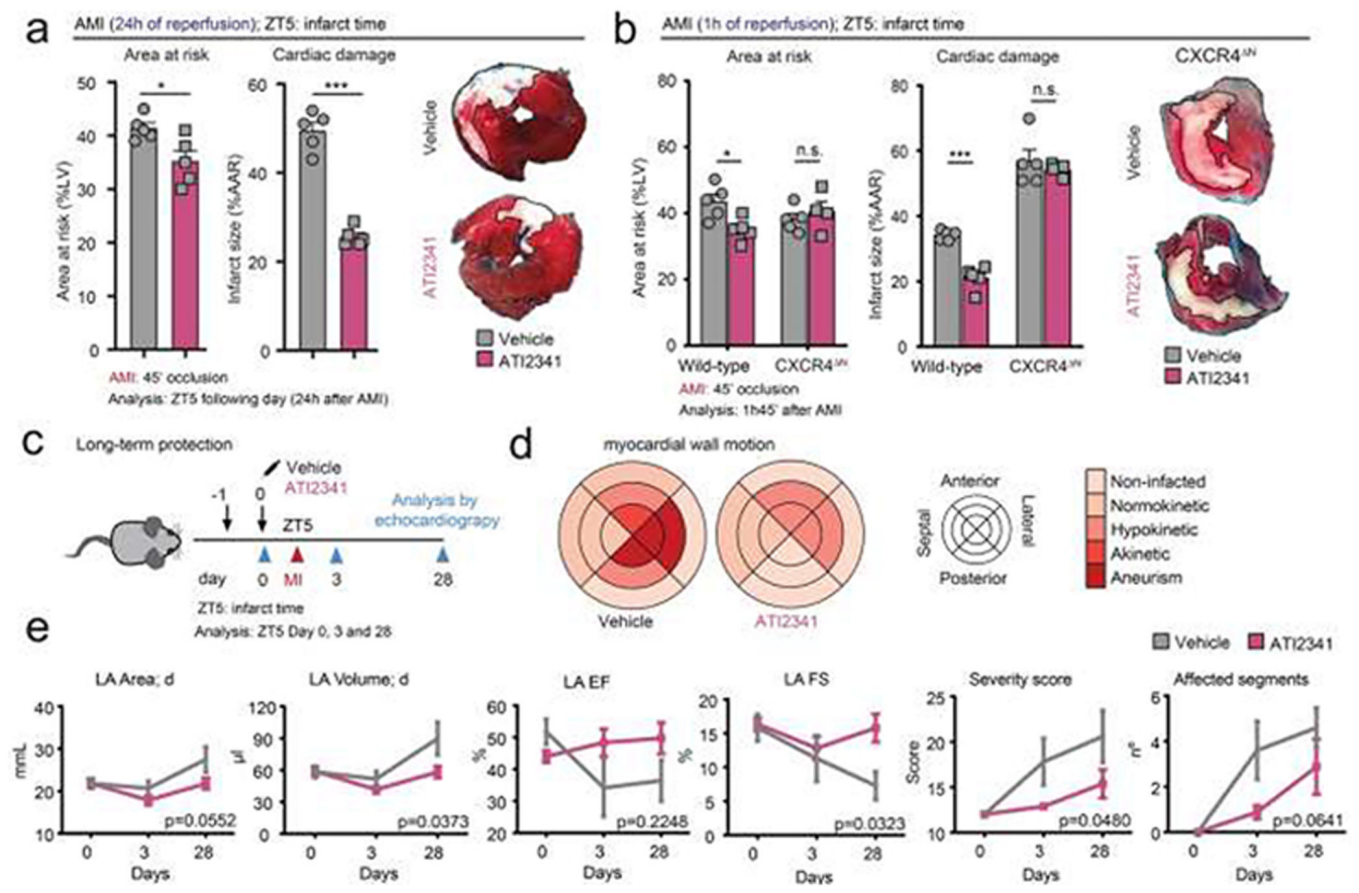
CXCR4 activation in neutrophils protects from ischemia-reperfusion injury. (a) Areas at risk (left graph) of the myocardium in vehicle- and ATI-treated mice infarcted at ZT5 and infarct sizes (right graph) after correction for areas at risk from the same animals subjected to MI and followed by 24h of reperfusion; n=5 mice per group. Representative images of infarcted hearts from vehicle- and ATI-treated mice at ZT5 after 24h of reperfusion. Dotted black lines highlight areas of dead myocardium. Analysis was performed 24h after infarction (the following day at ZT5). (b) Aras at risk (left graph) of the myocardium in vehicle- and agonist-treated WT and CXCR4^ΔN^ mice infarcted at ZT5 and infarct sizes (right graph) after correction for areas at risk from the same animals subjected to MI and followed by 1h of reperfusion; n=4-5 mice per group. Representative images of infarcted hearts from vehicle- and agonist-treated CXCR4^ΔN^ mice at ZT5. Dotted black lines highlight areas of dead myocardium. Analysis was performed 1h and 45min after infarction. (c) Experimental scheme to analyse cardiac function by echocardiography at different times after CXCR4 agonist treatment and permanent MI induced at ZT5. Analysis of cardiac function was performed at ZT5 at day 0 (basal, before infarction) and at day 3 and day 28 post-infarction. (d) Segmental wall motion of the myocardium. Colours reflect the different severities of segmental wall movement: non-infarction, normokinesis, hypokinesis, akinesis and aneurysm. (e) Selected cardiac parameters measured by echocardiography in vehicle and ATI2341-treated mice at different times. Left-atrium area (LA Area), Left-atrium volume (LA Volume), Left-atrium Ejection Fraction (LA EF), Left-atrium Fraction shortening (LA FS), severity score (Score) and number of myocardial affected segments (Affected segments) are shown. Some parameters were measured at diastole (d); n=5-8 mice per group. Data in (a-e) are from single experiments. Data are shown as mean ± SEM.; ***p<0.001; ns, not significant, as determined by unpaired t-test analysis (a) and two-way ANOVA (b, e).

We next studied the protective effect of CXCR4 agonism in the myocardial ischemia-reperfusion injury model, in which genetic interference with the neutrophil circadian clock conferred significant protection (Fig. 2a-c). Mice treated with ATI2341 displayed marked reductions in infarct size when induced at ZT5 and analyzed after 24 and 1h of reperfusion (Fig. 6a, b), without impacting neutrophil counts or any other hematological parameter, or organismal circadian patterns (Fig. 3i, j). Because multiple cell types express CXCR4, we examined whether the protection conferred by the agonist was mediated through specific signaling in neutrophils. To this end, we examined the response in mice with neutrophil-specific deletion of CXCR4 (CXCR4^ΔN^ mice). We found that these mice showed exaggerated myocardial injury after ischemia-reperfusion, supporting the basal protective relevance of this receptor against inflammatory injury (Fig. 6b). Importantly, the protective effect of the agonist was completely ablated in these mice (Fig. 6b), indicating that cardiac protection was mediated through specific targeting CXCR4 in neutrophils. To specifically examine whether the acute protection conferred by ATI2341 resulted in long-term improvement in cardiac function, we performed permanent ischemia and examined cardiac function in vehicle and ATI2341-treated mice up to 4 weeks after the ischemic injury (Fig. 6c). Longitudinal echocardiographic analyses revealed that cardiac wall motion was improved in ATI2341-treated mice, as reflected by reduced akinetic areas or aneurysms (Fig. 6d). Likewise, both systolic and diastolic functions of the cardiac tissue were preserved, as reflected by the reduced number of affected myocardial segments in the left ventricle (Fig. 6e). Thus, CXCR4 agonism effectively protects from inflammatory injury by targeting neutrophils and preserves both short- and long-term tissue function.

To further dissect the contribution of ATI2341-treated neutrophils to the circadian protection during sterile inflammation in the infarct model, we performed single-cell RNAseq analyses after myocardial infarction in agonist- and vehicle-treated mice (Fig. S3a-c). Uniform Manifold Approximation and Projection (UMAP) visualization allowed identification of seven distinct cell clusters (C0–C6) across our experimental groups (Fig. S3d). We focused on neutrophils (C2), as well as macrophages (C1), endothelial cells (C6) and fibroblasts (C5) for reference, from the total sorted cells from the infarcted hearts (Fig. S3d). While expression of ~6-10% genes was altered by the ischemia in these cells, we found virtually no genes affected by ATI2341 treatment (shown as volcano plots in Fig. S3g-h). We found more genes whose expression changed in neutrophils upon ATI2341 treatment, including *Cd177* and Cd101, which encode for proteins associated with inflammation during cardiac injury (Bouvain et al., 2023), and neutrophil maturation, respectively (Evrard et al., 2018) (Fig. S3g). Overall, however, these changes were modest, suggesting that the cardioprotective effect of ATI2341 was not mediated by transcriptional regulation of the neutrophils recruited to the infarcted myocardium.

### CXCR4 agonism relocates neutrophils to minimize collateral damage to host cells

The protective effects of the CXCR4 agonist on vascular inflammation and myocardial injury suggested that preservation of vascular integrity could be a common mechanism of protection. Unexpectedly, however, we found that vascular leakage, assessed by intravenous injection of the small dye Evans Blue, was not prevented in SCD mice after ATI2341 treatment, both at baseline and after TNFα challenge (Fig. S4a-b). This finding suggested that a reduction in intravascular occlusion and improved perfusion, rather than effects on the endothelium, was the mechanism of protection in these mice (Manwani and Frenette, 2013). Likewise, ATI2341 failed to prevent vascular leakage in wild-type mice upon lipopolysaccharide (LPS) treatment or after myocardial infarction (Fig. S4c-d), altogether indicating that preservation of vascular integrity was not the mechanism of protection. These findings aligned with our single-cel RNA sequencing (scRNAseq) data from the infarcted heart (Fig. S3a-d), which showed virtually no transcriptional impact of the agonist on endothelial cells from the infarcted hearts (Fig. S3h). Intriguingly, however, we found a modest upregulation of *Cxcl12* by endothelial cells after ATI2341 treatment, suggesting a potential feed-forward loop (Fig. S3h). Overall, these data suggested that the CXCR4 agonist induced its protective effect independently of transcriptional regulation or by direct effects on endothelial cells.

**Figure 7:**
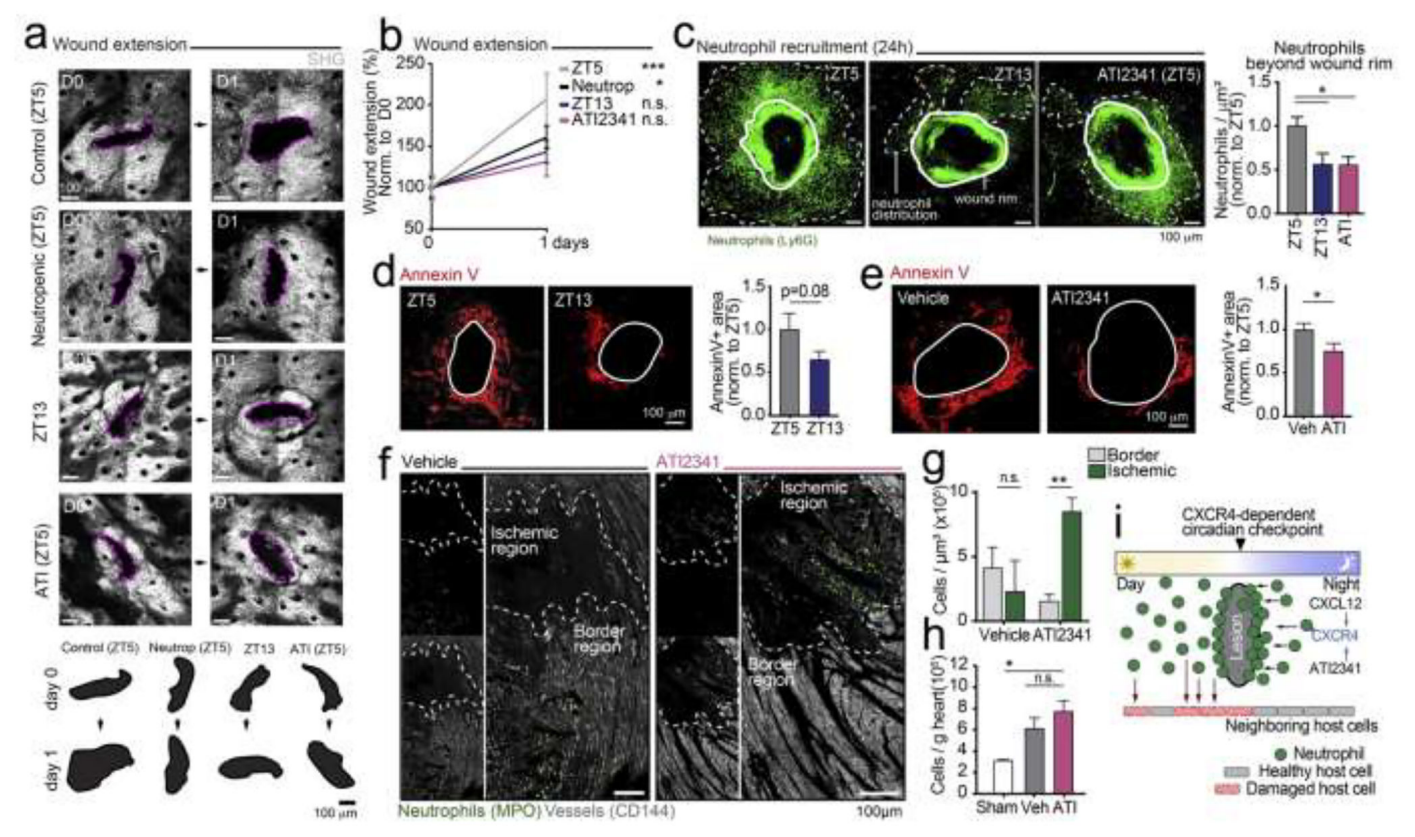
Circadian redistribution of neutrophils protects the surrounding tissue. (a) Representative imaging of skin wounds (at days 0 and 1; D0-D1) visualized by SHG acquired through multiphoton imaging control mice at ZT5 (WT), neutropenic mice at ZT5 (iDTR), control mice injured at ZT13 and control mice injured at ZT5 and treated with ATI2341. Dotted lines show the rim of skin wounds, also shown at bottom. (b) Quantification of the increase in wound size from D0 to D1 (24h after injury) in the indicated mice; n=3-4 mice per group, 2-5 wounds per mouse. (c) Whole-mount imaging of neutrophils (left) in and around skin wounds of wild-type (C57BL6 or Ly6G^Tomato^ mice) injured at ZT5 and ZT13, and ATI2341-treated mice at ZT5, quantified in the bars at right; n=3-6 mice per group (1-2 wounds per mouse). Solid white lines show the wound limits by the neutrophil rim, and dotted white lines show the distribution of neutrophil around the wounds. (d, e) Representative confocal images (left) and quantification (right) of annexin V signal around skin wounds from mice injured at ZT5 or ZT13 ((d); n= 6-8 mice per group, 3-5 wounds per mouse), or around skin wounds from vehicle- or ATI-treated mice ((e); n= 6-7 mice per group, 2-4 wounds per mouse). (f, g) Representative images (f) and quantification (g) of neutrophil distribution in the infarcted myocardium of control and ATI2341-treated mice; n= 3 mice per group, 2 ischemic and 4 border regions quantified per mice. The ischemic areas are identified by lack of vascular staining (VE-Cadherin) and are delimited by white dotted lines. Neutrophils in and outside this area were identified by MPO staining. (h) Neutrophil numbers in the left ventricle of infarcted hearts from Sham (placebo surgery), vehicle- and ATI2341-treated mice; n=3-4 mice per group, 2-4 regions per mouse. (i) Summary cartoon showing neutrophil redistribution into wounds, driven by CXCR4 signaling, that occurs naturally at nighttime and can be induced by CXCR4 agonism, resulting in reduced collateral damage to the surrounding tissue. Data in (a-b and g) are from single experiments. Data in (c and d) are pooled from three experiments. Data in (e) are pooled from four experiments. Data in (h) are representative of two experiments. Data are shown as mean ± SEM. *p<0.05; **p<0.001; ns, not significant, as determined by one-way ANOVA (c, h), unpaired t test (d, e) and two-way ANOVA (b, g).

We therefore hypothesized that the protective mechanism of CXCR4 agonism during ischemic injury might occur once neutrophils had entered the inflamed tissue. To examine this possibility, we used a simple model of focal injury induced by needle puncture across the full thickness of the ear skin (Fig. S5a). This model elicits rapid and local recruitment of neutrophils and allows high-resolution, quantitative imaging in a two-dimensional tissue (Ng et al., 2011), and therefore allowed for simpler exploration of mechanisms at play in the infarcted heart. Confocal imaging of skin wounds induced at ZT5 in Ly6G^Tomato^ reporter mice (Hasenberg et al., 2015) revealed rapid infiltration of neutrophils, which accumulated around the wounds by 24 hours (Fig. S5b) and mediated the enlargement of the lesions, as their depletion prevented a marked increase in wound size (Fig. 7a-b). To examine the impact of diurnal time and clock inhibition in this model, we imaged skin lesions elicited at different diurnal times, or after treatment with the CXCR4 agonist. Notably, we found that skin wounds did not increase in size when induced at ZT13, or when induced at ZT5 after treatment with ATI2341 (Fig. 7a-b), thus mimicking our observations in the myocardial infarction model (Fig. 1 and Fig. 6). We also noticed that neutrophils distributed in a relatively large area beyond the immediate wound rim when the wounds were generated at ZT5. Strikingly, when wounds were induced at ZT13 the area covered by neutrophils was substantially reduced, such that the neighboring unaffected tissue was spared from the presence of neutrophils (Fig. 7c). ATI2341 treatment recapitulated this effect in wounds generated at ZT5, which now showed a redistribution of neutrophils more proximal to the wound rim, mimicking the distribution seen at ZT13 (Fig. 7c). This effect was not caused by reductions in neutrophil infiltration, since the number of cells recruited to the lesions was similar across all groups, as determined by flow cytometry (Fig. S5c). Importantly, analysis of the wounds in CXCR4^ΔN^ mice revealed that CXCR4 signaling in neutrophils was necessary for their redistribution around the wounds induced by ATI2341, because the effect of the agonist was lost in these mice (Fig. S5d).

We speculated that the broad distribution of neutrophils recruited around the initial wound could cause significant damage to the surrounding host tissue through non-specific cytotoxic activity. To assess if this was the case, we searched for evidence of cell death around wounds by staining fresh skin explants with fluorescently conjugated Annexin V to probe for the presence of apoptotic cells. Consistent with our prediction, we found areas of prominent apoptosis that extended 100 μm or more beyond the rim of wounds generated at ZT5 (Fig. 7d). These areas of cell death were reduced in neutropenic mice (Fig. S5e) or when the wounds were generated at ZT13 (Fig. 7d), indicating that they reflected the cytotoxic activity of neutrophils that accumulated around the initial lesion. Notably, ATI2341 also elicited a marked reduction in the area of cell death around the wounds (Fig. 7e) and we confirmed this observation using the Terminal deoxynucleotidyl transferase dUTP nick-end labeling (TUNEL) assay to detect DNA breaks associated with cell death (Fig. S5f). Together, these data reveal a circadian checkpoint that relocates neutrophils away from healthy host cells around lesions at nighttime, reducing cell death of the neighboring tissue. It also shows that this protective effect can be mimicked by activating neutrophils via CXCR4.

To examine if this mechanism underlay the protection of the myocardial tissue after ischemia-reperfusion elicited by the CXCR4 agonist, we performed whole-mount imaging of heart sections from vehicle- or ATI2341-treated mice 24h after surgery. Consistent with the skin wound model, we found that over half of the recruited neutrophils were positioned in the border zone outside the ischemic lesion in control mice, and ATI2431 repositioned most neutrophils away from the border zone and inside the ischemic lesion (Fig. 7f-g), without altering the total number of neutrophils that infiltrated the tissue (Fig. 7h). Thus, CXCR4 agonism relocates neutrophils away from the healthy neighboring tissue, a circadian phenomenon that is naturally active at night and prevents indiscriminate tissue death (Fig. 7i).

**Figure 8:**
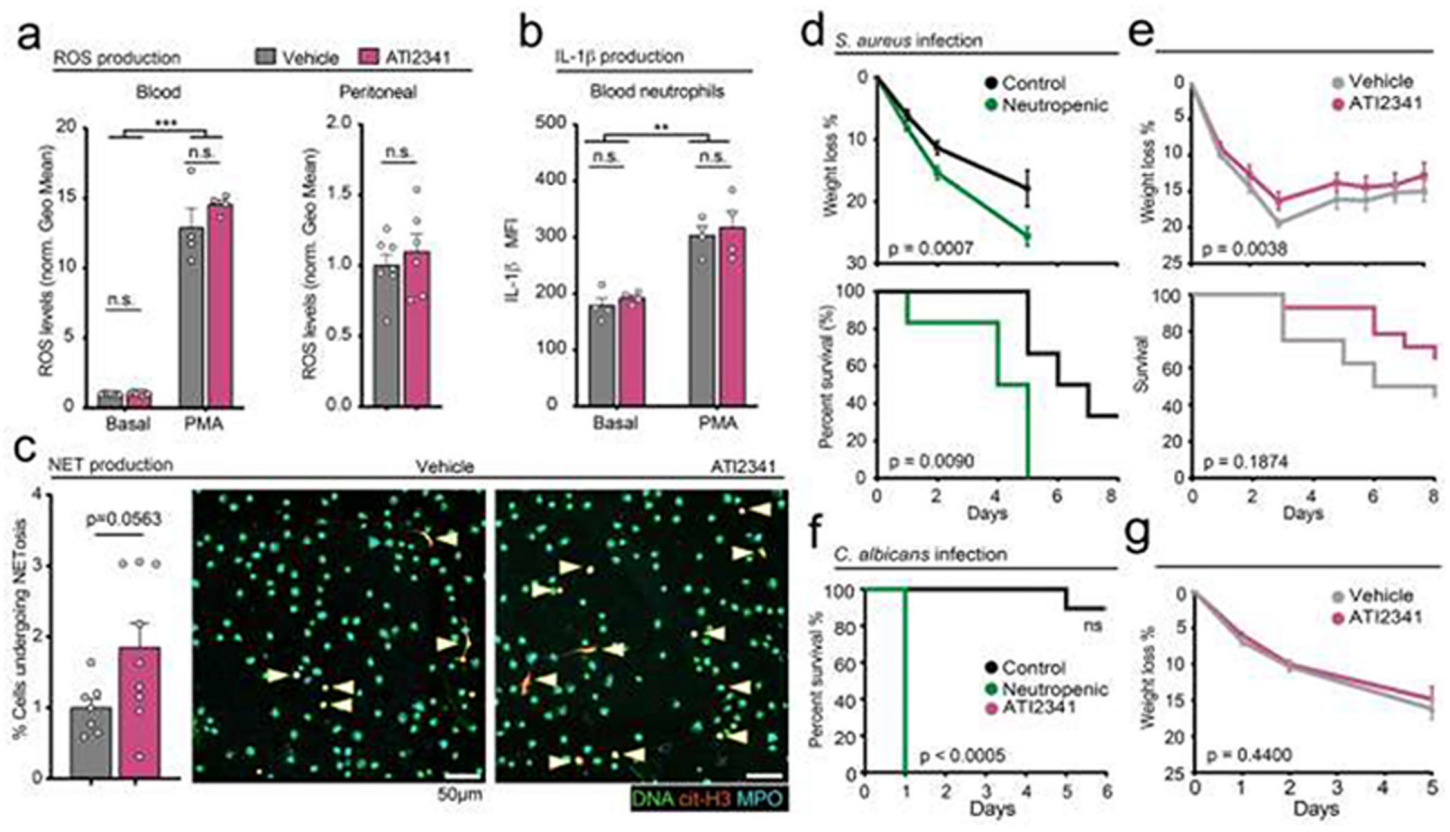
ATI2341 does not interfere with antimicrobial responses. (a) ROS production measured by DHE oxidation as determined by flow cytometry in blood neutrophils from vehicle- and ATI2341-treated mice after *ex vivo* stimulation with vehicle or PMA (left) and in peritoneal neutrophils after *in vivo* administration of zymosan (right); n= 4-7 (left) and 6-7(right) mice per group. (b) Expression of IL-1β in blood neutrophils from control and ATI2341-treated mice after *ex vivo* stimulation with vehicle or PMA as determined by flow cytometry; n=4 mice per group. **(c)** *Ex vivo* NET-formation assay with sorted blood neutrophils at daytime (ZT5) stimulated with PMA from control and ATI2341-treated mice. NETs were quantified (left) by triple colocalization of cit-H3, DNA and MPO in confocal micrographs (right); n=7-9 mice per group. **(d)** Weight-loss (up) and survival (bottom) curves of control and neutropenic mice (Mcl1^fl/fl^) infected with *S. aureus* at ZT5 and monitored for 8 days; n=6 mice per group. **(e)** Weight-loss (up) and survival (bottom) curves of vehicle- and ATI2341-treated mice infected with S. aureus at ZT5 and monitored for 8 days; n=14-16 mice per group **(f)** Survival curves of neutropenic (Mcl1^fl/fl^), vehicle- and ATI2341-treated mice infected with C. albicans at ZT5 and monitored for 5 days; n=3-10 mice per group. **(g)** Weight-loss curves of vehicle- and ATI2341-treated mice infected with *C. albicans* at ZT5; n=10 mice per group. Data in (b and d) are from single experiments. Data in (a, e, f and g) are pooled from two experiments. Data in (c) are pooled from three experiments. Except for survival curves, data are shown as mean ± SEM. *p<0.05; **p<0.01; ***p<0.001; ns, not significant, as determined by two-way ANOVA (a (left), b, d (up), e (up) and g), unpaired t test analysis (a (right), c) and log rank test (d (bottom), e (bottom) and f).

Because neutrophils provide the first line of defense against invading microbes, we asked whether the protective effects of the CXCR4 agonist by redistributing neutrophils in the affected tissue could be detrimental during infections. *Ex vivo* analyses revealed that neutrophils from ATI2341-treated mice produced normal levels of reactive oxygen species (ROS) and IL1β (Fig. 8a-b), two important mediators of antimicrobial defense (Burn et al., 2021). Likewise, blood neutrophils from ATI2341-treated mice produced neutrophil-extracellular traps (NETs) normally (Fig. 8c), altogether suggesting that the agonist did not interfere with the cytotoxic and pro-inflammatory activity of neutrophils. Finally, to test the antimicrobial response upon CXCR4 activation, we challenged vehicle- and ATI2341-treated mice with *C. albicans* or with *S. aureus* and monitored their weight and survival over the following five to eight days, respectively. We found that ATI2341 did not interfere with the defense against these common commensals and, in fact, showed mild but significant improvement in the case of *S. aureus* (Fig. 8d-g). This contrasted with neutrophil depletion, which also alleviated the inflammatory response (Fig. 1b) but dramatically worsened the outcome of mice to the bacterial and fungal infections (Fig. 8d, f). Thus, the protective effect of CXCR4 agonism during sterile inflammation does not compromise antimicrobial responses, suggesting that targeting this circadian neutrophil checkpoint driven by CXCR4 may be an attractive therapeutic target against the devastating impact of uncontrolled inflammation in human health.

## Discussion

Circadian oscillations are an anticipatory phenomenon that optimizes the organismal response to environmental changes. For the immune system, these circadian rhythms provide a means to regulate the magnitude and quality of antimicrobial and inflammatory responses (Scheiermann et al., 2018) by controlling multiple parameters in the cell, from proliferation and gene expression to cell topology and migration (Ovadia et al., 2023; Scheiermann et al., 2018). Ultimately, these circadian oscillations impact both the timing and outcome of infection and sterile inflammation (Wang et al., 2022). For this reason, defining the molecular switch that controls the transition from an aggressive to a more permissive response is key for harnessing this natural mechanism for human health. Here, we build on our previous studies on circadian mechanisms controlling neutrophil function (Adrover et al., 2020, 2019) to show that activation via CXCR4 provides such a switch for neutrophils and demonstrate that pharmacological delivery of a synthetic agonist for this receptor induces the transition of neutrophils to a night-like, permissive state that alleviates the inflammatory response without interfering with antimicrobial defense. This therapeutic strategy provides an advantage when compared with other approaches that target neutrophil function or numbers, which compromise the capacity of the host to control infections (Navarini et al., 2009; Neth et al., 2005) or to promote wound healing (Lörchner et al., 2015; Moutsopoulos et al., 2017; Ng et al., 2011; Peiseler and Kubes, 2019). Our study additionally shows that the neutrophil-intrinsic clock is necessary to dictate the oscillatory patterns of inflammatory injury, and provides mechanistic insights by showing that, in tissues, protection at specific times of day is accomplished by relocating these cytotoxic cells away from the healthy areas of the tissue.

While our findings may have direct implications for the clinic, here we highlight several new insights into the mechanisms of neutrophil-driven inflammation. First, we show that the basal activation state of neutrophils, controlled by an intrinsic circadian clock, dominates over cell number in determining the outcome of inflammation. Genetic inhibition of the circadian clock specifically in neutrophils (using Bmal1^ΔN^ mice) alleviates the damage to ischemic tissues despite similar or even higher numbers and circadian dynamics of circulating neutrophils as in control mice. Consistently, this and previous studies (Adrover et al., 2019) show that although challenges at nighttime elicit milder inflammatory responses in mice, the migration of neutrophils to sites of inflammation is equal or even more efficient at this time (Adrover et al., 2019), resulting in overall similar numbers of recruited neutrophils to these sites. This is despite multiple studies associating the number of circulating neutrophils and neutrophil-to-lymphocyte ratios with the outcome of disease in humans (Buonacera et al., 2022; Coller, 2005), which we propose may reflect the presence of other subclinical conditions that activate additional inflammatory mechanisms. A recent study has shown altered sleep patterns after myocardial ischemia (Huynh et al., 2024), raising the intriguing possibility that disease can reciprocally reset the diurnal patterns of neutrophil activity, potentially impacting subsequent inflammatory insults.

Second, our study reveals a dissociation between the inflammatory and antimicrobial activities of neutrophils. Treatment with the CXCR4 agonist blunts the inflammatory response without interfering with antimicrobial defense. In fact, the improved response to *S. aureus* infection upon ATI2341 treatment may be associated with the preferred migration of neutrophils to areas where bacteria nest inside the host, mirroring the behavior seen in the sterile models of local inflammation (skin wounds or cardiac ischemia), resulting in more efficient control of the pathogens. These findings highlight the importance of the microanatomical location of neutrophils rather than just entry into the affected tissue. In this regard, we propose that the distribution of neutrophils beyond the limits of the wound at daytime may be evolutionarily beneficial by allowing neutrophils to scout other areas in search of disseminated microbes. It is this exploratory behavior, away from the initial lesion and in contact with healthy tissue cells, that ultimately appears to extend the initial lesion and is at the core of the inflammatory injury elicited by neutrophils (Silvestre-Roig et al., 2020).

Finally, the finding that CXCR4 agonism protects from vascular occlusion allowed us to discover that the “behavioral” properties of intravascular neutrophils are under circadian regulation. We show that the pathogenic subpopulation of B3 neutrophils (Crainiciuc et al., 2022) is largely restricted to daytime and that, in the context of SCD, these cells are responsible for the capture sRBC leading to vaso-occlusive episodes (Hidalgo et al., 2009; Torres and Hidalgo, 2023; Zhang et al., 2016). Whether the effect is driven by direct signaling and inhibition of the adhesive activity of intravascular neutrophils and/or selective elimination of these pathogenic cells from circulation remains an open question. It is additionally possible that cross-inhibition of CXCR2 signaling by CXCR4 (Martin et al., 2003) alters the migratory pattern and activation of certain types of neutrophils. In addition, the lack of effect of the CXCR4 agonist on vascular leakage suggests that this axis only targets some activities of neutrophils, for example it inhibits their capacity to capture circulating sRBCs but preserves their antimicrobial activity.

Despite these insights, our study retains limitations and opens areas for future exploration. For instance, better characterization of the pharmacokinetics, pharmacodynamics and dose-dependent response of ATI2341 is needed to assess the clinical value of this or similar compounds. Likewise, the systemic treatment with this compound is likely to have off-target effects through activation of CXCR4 in other lineages that express this receptor, including immune, vascular, and parenchymal cells (Bianchi and Mezzapelle, 2020; Döring et al., 2017), Our analyses, however, suggest that the effects in other lineages are minimal, at least at the transcriptional level. Further, we present evidence that signaling via CXCR4 in neutrophils is necessary for the beneficial effects of the agonist seen during skin and cardiac injury. Regardless, it will be important to define whether signaling through neutrophils is also sufficient to elicit tissue protection because CXCR4 activation has been shown to mediate the mobilization of immature cells with anti-inflammatory effects (Blum et al., 2009) and to elicit beneficial effects on endothelial and mesenchymal cells (Chi et al., 2021; Jin et al., 2013; Li et al., 2021). Although these effects require longer times than those seen in the context of acute inflammation, they may contribute to the long-term benefits to the injured tissue. Another open question is how neutrophils achieve their distinct distribution in the wounded tissue at night or upon activation via CXCR4; it is likely that cross-regulation of other receptors involved in adhesion and migration across the different microenvironments and chemokine gradients present in the tissue mediate this phenomenon. Additionally, it will be important to define the effect of CXCR4 agonists in other pathophysiological contexts in which redistribution of neutrophils may have detrimental effects, such as the context of solid tumors in which accumulation of pro-tumoral neutrophils or neutrophil-derived products, such as NETs and leukotrienes (Albrengues et al., 2018; Teijeira et al., 2020; Wculek and Malanchi, 2015), can contribute to disease progression. Finally, it is also important to consider that the recruitment of neutrophils to peripheral tissues following CXCR4 signaling may serve functions beyond the control or aggravation of local inflammation. This possibility is supported by recent findings in monocytes showing that their recruitment to the brain after myocardial infarction promotes restorative sleep and limits sympathetic output (Huynh et al., 2024).

In sum, we have identified a ligand-receptor pair that provides a circadian checkpoint for neutrophil activation and distribution and is, in this regard, reminiscent of those controlling the effector functions of T lymphocytes (Wei et al., 2018). While full characterization and identification of the off-target effects of CXCR4 agonists are ongoing, we propose that this axis provides an attractive approach to therapeutically modulate the function of neutrophils, the largest army of cytotoxic cells in our body.

## Materials and Methods

### Experimental mice and *in vivo* treatments

All experiments were performed in 7-to 16-week-old male mice in a C57BL/6 genetic background. Bmal1^ΔN^ and CXCR4^ΔN^ mutant mice with neutrophil-specific deficiency of Bmal1 and CXCR4, respectively, have been previously described (Adrover et al., 2019). CXCR4^WHIM^ mice with a hyper-signaling form of CXCR4 have been also described and were used in heterozygosis both as experimental mice and as donors to generate bone marrow chimeras (Balabanian et al., 2012). For bone marrow transplant experiments, WT donor bone marrow came from mice expressing DsRed under the control of the β-actin promoter (DsRedTg) (Vintersten et al., 2004). For intravital imaging experiments, we used LyzM^GFP^ mice (Faust et al., 2000) and generated WHIM;LyzM^GFP^ mice by crossing CXCR4^WHIM^ mice with the reporter knock-in line LyzM^GFP^. Both were used in heterozygosis to prevent gene deletion. Constitutively neutropenic mice (Mcl1^ΔN^) were generated by crossing LysM^Cre^ with Mcl1^tm1Ywh^ (Mcl1^fl/fl^) mice (Csepregi et al., 2018) and were used as a model of neutropenia (referred in the text as neutropenic). In some experiments (Fig. 7a, b) we used mice inducible for acute neutropenia by diphtheria toxin (DT) injection (iDTR) previously generated by crossing Tg(S100A8-cre,-EGFP)^1Ilw^ (Mrp8^Cre^) with C57BL/6-Gt(ROSA)26Sor^tm1(HBEGF)Awai/J^ (Rosa26iDTR) mice, as characterized in previous studies (Ballesteros et al., 2020). For confocal microscopy experiments, Ly6G^tdTomato^ mice (Hasenberg et al., 2015) were used to identify granulocytes by tomato expression. In the SCD model, we used Berkeley SS mice (Pászty et al., 1997) as donors for bone marrow transplantation. Mice were kept in a specific pathogen-free facility at Centro Nacional de Investigaciones Cardiovasculares (CNIC) under a 12h light/12h darkness schedule (lights on at 7am, off at 7pm), with water and chow available ad libitum. All procedures with backcrossed mice were controlled using littermate control mice. No specific randomization method was followed in this study. All experimental procedures were approved by the Animal Care and Ethics Committee of CNIC and the regional authorities (PROEX 101/18 // PROEX 194.2/21// PROEX 059.524).

### Neutrophil depletion

For neutrophil depletion, we injected mice intraperitoneally with 100 µg of anti-mouse Ly6G antibody (1A8 clone; BioXCell) at 48h and 24h prior to analysis as reported (Casanova-Acebes et al., 2013) or with 100 µg of a cocktail combination of anti-mouse Ly6G antibody (clones 1A8; BioXCell; West Lebanon, NH) and anti-rat Kappa light chain antibody (clone MAR 18.5; BioXCell; West Lebanon, NH) at 48h, 24h and 2h prior to myocardial ischemia/reperfusion (I/R) (24h circadian experiment), resulting in >80% reduction in neutrophil blood counts. In both cases, we administered an anti-rat IgG2a isotype antibody (clone 2A3; BioXCell) to the control group. Lymphocyte and monocyte counts were not affected by these treatments.

### Platelet depletion

For platelet depletion, we injected mice intraperitoneally with 50µl of rabbit anti-platelet serum (Accurate Chemical) diluted in 200 µl of PBS 24h before intravital surgery with >95% depletion efficiency. White blood cells (WBC) and red blood cells (RBC) counts were not affected by this treatment.

### CXCR4 agonist treatment

To target CXCR4 in neutrophils, we treated mice intraperitoneally with the CXCR4 commercial agonist ATI2341(Tocris) diluted in PBS at a dose of 1mg/kg at two different times (ZT13: day before and ZT2: experiment day). Control (vehicle) mice were injected with the same volume of PBS.

### Acute and permanent myocardial infarction

To study the effect of ZT, genes or treatments on myocardial infarct size, we subjected 8-to 14-week-old male mice to 45 min occlusion of the left anterior descending (LAD) coronary artery followed by 1h or 24h of reperfusion (for infarct size). The I/R procedure was performed as previously described (Adrover et al., 2019; García-Prieto et al., 2017). In brief, fully anesthetized animals were intubated, and temperature controlled throughout the experiment at 37.5 °C to prevent hypothermia-driven cardioprotection. Then, we performed thoracotomy and ligation of the LAD coronary artery with a nylon 8/0 monofilament suture for 45 min, and monitored the electrocardiogram (MP36R, Biopac Systems) to confirm total coronary artery occlusion (ST-segment elevation) throughout the 45 min of ischaemia. At the end of the ischemia, the chest was closed, and animals were kept with 100% O_2_ and analgesized with buprenorphine (subcutaneous injection, 0.1 mg/kg). For quantification of infarct size, we re-anesthetized and re-intubated mice, and the LAD coronary artery was re-occluded by ligating the suture in the same position as the original infarction. Animals were then euthanized and 1 ml of 1% dilution of Evans Blue dye (Sigma) was infused intravenously to delineate the area at risk (AAR: myocardium lacking blood flow, i.e. negative for blue dye staining). Then, the left ventricle (LV) was isolated and cut into transverse slices (6 slices, 1-2mm thick slices per LV), and both sides were imaged. To delineate the infarcted (necrotic) myocardium, we incubated the slices in triphenyltetrazolium chloride (TTC, Sigma) at 37 °C for 10 min. We re-photographed and weighed the slices, and regions negative for Evans Blue staining (AAR) and for TTC (infarcted myocardium) were quantified using ImageJ (NIH, Bethesda, MD). Percentage values for AAR and infarcted myocardium were corrected for weight (mg) independently for each slice. Absolute AAR and infarct size were determined as the mg:mg ratio of AAR:LV and infarcted myocardium:AAR, respectively. Outcome assessment was performed blind to condition (mouse type, zeitgeber time or treatment).

For some groups, we also performed MRI for analysis of cardiac function after 7 days of I/R. Briefly, mice were anaesthetized with isoflurane (2% in oxygen, 0.5 L/min) and were monitored for core body temperature and respiration rate using a specific monitoring system (SA Instruments Inc., New York, NY). In vivo cardiac images were acquired using a 7T magnet from Agilent (Agilent Varian, Palo Alto, USA) controlled by an Avance NEO Spectrometer (Bruker, Ettlingen, Germany). An IntraGate FLASH sequence was used with a combination of a 112/86mm resonator and a surface heart array coil (Bruker, Ettlingen, Germany). Cardiac four-chamber and left two-chamber views were acquired and used to plan the short axis sequence. Mice were imaged with the following parameter settings: number of slices, 11-14; slice thickness, 0.8 mm; data matrix size, 256x256; field of view, 25x25 mm2; movie frames, 12; oversampling, 100; TE, 1.58 ms and TR, 8.5 ms; flip angle, 20º. Cine Magnetic Resonance (CMR) images were exported to Dicom format and analysed with Segment software (Mediso AB v.4.1.0.1 R14284b). The short-axis dataset and cine modus short-axis view were analysed quantitatively by manual detection of RV and LV endocardial borders in end diastole and end systole without exclusion of papillary muscles and trabeculae to obtain ejection fraction (%EF).

For permanent myocardial infarction, we subjected 8-to 14-week-old male mice to permanent occlusion of the left anterior descending (LAD) coronary artery to measure long-term cardiac function. We measured cardiac function at basal time and days 3 and 28 post occlusion by echocardiography using a Vevo 2100 Ultra High Frequency ultrasound (Visualsonics Inc.) with support of the Advanced Imaging Unit of CNIC. A base apex electrocardiogram was continuously monitored through 4 leads connected to the ultrasound machine and isoflurane delivery was adjusted to maintain the heart rate in 450 ± 50 bpm. Normothermia was maintained placing mice in a heating platform. Images were recorded and transferred to a computer for posterior blinded analysis using the Vevo 2100 Workstation software. Parasternal standard 2D and M-mode (MM) long and short axis views (LAX and SAX view, respectively) were acquired to assess left atrium (LA) and ventricular (LV) systolic function and dimensions (LA-Volume;d and LA-Area;d). LA ejection fraction (LA-EF), LA fraction shortening (LA-FS), interventricular septum thickness (IVS;d) and end-diastolic posterior wall thickness (LVPW,d) were subsequently calculated. A 2D apical view was used to evaluate diastolic dysfunction, using pulsed-wave (PW) Doppler, to estimate mitral valve inflow pattern. Early and late diastolic velocity peak waves (E and A, respectively) were measured, and the E/A ratio calculated.

### Generation of bone marrow chimeric mice

To analyze mutant neutrophils in the same physiological context as WT neutrophils, we generated BM chimeras. Donor bone marrow cells were harvested from WT^DsRed^ and mutant mice by flushing the femur into PBS 1x. We then injected 10^6^ bone marrow nucleated cells containing an equal mix of wild-type and mutant cells into recipient male C57BL/6 mice sub-lethally irradiated (6.5 Gy, two split doses, 4h apart). 4-6 weeks after transplantation, we evaluated engraftment and chimerism of recipient animals by analyzing the percentage of wild-type and mutant leukocytes in peripheral blood before further experimentation.

Chimeric sickle cell mice were generated by transplantation of bone marrow cells (obtained as described above) from transgenic Berkeley mice (homozygous for the alpha-globin null allele, homozygous for the beta-globin null allele and carrying the sickle transgene, Hba^0/0^ Hbb^0/0^ Tg(Hu-miniLCRα1^G^γ^A^γδβ^S^)) into recipient male C57BL/6 mice lethally irradiated (12 Gy, two split doses), as previously described (Turhan et al., 2002). BM reconstitution efficiency was analyzed in recipient mice 3 months after transplantation (the minimum time for full replacement of circulating RBCs with those of donor origin in lethally irradiated mice), and fully reconstituted mice (>97% of sickle human hemoglobin) were used for experiments (referred to as SCD mice).

### Infection models

To determine susceptibility to infection, mice were intravenously infected with 1.25x10^5^ colony-forming units (CFUs) of *Candida albicans* (SC5314 strain) or with 3.5x10^7^ CFUs of *Staphylococcus aureus* (RNU4220 strain) and daily monitored for weight loss and general health following the institutional guidance. In the *C. albicans* infection model, kidney fungal burden was determined at day 6 post-infection by plating serial dilutions of organ homogenates on Yeast Extract Peptone Dextrose (YPD, Sigma) plates. CFUs were counted after growth for 48 h at 30 °C. Survival times were defined as the time from fungal or bacterial injection until death or weight loss higher than 20%, addressed up to 6 or 8 days, respectively. During fungal and staphylococcal infection, blood was collected at initial and/or final time points for hematology. Previously to infection, *Candida albicans* was grown in YPD medium at 30ºC and *Staphylococcus aureus* in Brain Heart Infusion (BHI, Sigma) medium at 37°C for 4 hours to an optical density at 600 nm (OD600) of 0.8. The culture was centrifuged at 1800 rpm for 5 min and the pellet resuspended in PBS 1x at the indicated concentration for injection.

### Vascular permeability assay

For vascular permeability assays, 200 μl of a 0.5% solution of Evans Blue prepared in sterile PBS was injected intravenously into mice 4h after LPS injection (1mg/kg, intraperitoneally) for endotoxemic mice, TNFα administration (0.5μg/mice, intraperitoneally) for SCD mice or after 45 min of ischemia for infarcted mice. After 15 min, we euthanized the mice and extracted tissues for weight and leakage. Tissues were submerged in 0.5 ml of formamide and incubated at 55ºC for 24h. After incubation, tissues were removed and tubes centrifuged for 5 min at 645 g. Finally, we measured supernatants for absorbance at 610nm using an xMark Microplate Spectrophotometer (BioRad) or a BioTek Synergy H4 Hybrid (Fisher) plate reader, and absorbance was corrected by the weight of the tissue analysed.

### Ear wound model

Mice were anesthetized by intraperitoneal injection of 7.5% ketamine: 5% xylazine mixture (10μl/g). Ears were held gently in place with forceps and pierced 4-5 times with a 29G needle from the ventral side below. The pierced sites were chosen approximately 3 to 6 mm from the tip of the mouse ear and with a separation among them around 6 to 9 mm. After 1h or 24 hours, mice were euthanized with CO_2_ and carefully perfused with 10 ml of PBS. Both ears were harvested and fixed with 4% paraformaldehyde (PFA) overnight or processed immediately for subsequent analysis.

### Metabolic cages

Oxygen consumption rates, carbon dioxide release, spontaneous locomotor activity and food consumption were monitored for individually housed mice using the OxyletPro Physiocages (Panlab). A period of 3-7 days of acclimatization was taken before the actual recordings. Data was collected at 2-minute intervals. The light schedule in the metabolic cages was maintained as in the animals’ home cages (12h light/12h darkness schedule).

### Flow cytometry and cell sorting

Cytometric analyses were performed using a Sony SP6800 Spectral Analyzer (Sony Biotechnology), Canto 3L (BD Biosciences) or FACSymphony (BD Biosciences). Cell sorting experiments were performed using a FACS Aria cell sorter (BD Biosciences). In all cases we obtained purities > 95%. All the analyses were performed at the Celomic Unit at Centro Nacional de Investigaciones Cardiovasculares (CNIC). The FlowJo software (FlowJo LLC) was used to analyze the data.

Primary antibodies used for flow cytometry assays during this project include FITC-conjugated antibodies to mouse CD11b (BD Biosciences, clone M1/70), CD62L (eBioscience, clone MEL-14), Ly6C (eBioscience, clone HK1.4), Ly6G (eBioscience, clone 1A8) and CD45R (BD Biosciences, clone RA3-6B2); PE-conjugated antibodies to mouse CD11b (Tonbo Biosciences, clone M1/70), CXCR4 (BD Biosciences, clone 2B11), CCR2 (R&D Systems, clone RA3-6B2) and Ly6G (Biolegend, clone 1A8); APC-conjugated antibodies to mouse CXCR4 (eBioscience, clone 2B11), IL1b (eBioscience, clone NJTEN3), Ly6B.2 (Miltenyi, clone REA115) and SiglecF (BD Biosciences, E50-2440); PE-Cy7-conjugated antibody to mouse CD101 (Invitrogen, clone Moushi101) and CD4 (Tonbo Biosciences, clone RM4-5); BV510-conjugated antibody to mouse CD11b (Biolegend, clone M1/70); APC-Cy7-conjugated antibody to mouse CD45 (Biolegend, clone 30-F11), CD8a (Tonbo Biosciences, clone 53-6.7) and CD45R (BD Biosciences, clone RA3-6B2); PerCPCy5.5-conjugated antibody to mouse CXCR2 (Biolegend, clone SA044G4) and CD45 (Biolegend, 30-F11); AF647-conjugated antibody to mouse Ly6G (Biolegend, clone 1A8); Dylight 450-conjugated antibody to mouse Ly6G (clone 1A8, BioXcell conjugated in house), AF488-conjugated antibody to CD3e (BD Biosciences, clone 145-2C11), BV711-conjugated antibody to mouse Ly6C (Biolegend, clone HK1.4), BV650-conjugated antibody to mouse CD64 (BD Biosciences, clone X54-5/7.1) and BUV737-conjugated antibody to mouse Ly6G (BD Biosciences, clone 1A8). We also used Dihydrorhodamine 123 (DHR123, Invitrogen) to measure ROS production by flow cytometry.

For some experiments, we measured absolute numbers of cells present in tissues. Truecount beads (Truecount absolute counting tubes, BD) were prepared at a concentration of 10,000 beads per ml in PEB buffer (PBS 1x, EDTA 0.5M, FBS 0.5%) containing DAPI 1/10000 (Life Technologies). 300-400 μl of this PEB/DAPI buffer containing beads were added to single cell suspensions stained for flow cytometry. Then cell numbers were calculated based on the number of beads per tube and corrected by the weight or volume of tissue analysed.

### Quantification of neutrophil numbers in circulation

To quantify blood neutrophil counts in control and neutrophil-depleted mice at different circadian times, mice were bled at the indicated times and 30 μl of blood cells were stained with BV510-conjugated anti-CD11b (1:200), FITC-conjugated anti-Ly6C (1:200) and APC-conjugated anti-Ly6B (1:200) for 15 min at 4 ºC. Neutrophils were identified as CD11b^+^Ly6C^med^Ly6B^+^, and numbers were estimated with PEB/DAPI containing True Count Beads (BD Biosciences). For additional determination of blood absolute cell numbers given as cells per ml in mutant or agonist-treated mice, we also used automated hemocounters (Abacus Junior, Diatron; ABX Pentra 80, Horiba). For quantification of lymphoid and myeloid cells in the blood of mutant mice at different circadian times, 30 μl of blood cells were stained with BV510-conjugated anti-CD11b, PE-conjugated anti-Ly6G, AF488-conjugated anti-CD3e, APC Cy7-conjugated anti-CD45R and PerCPCy5.5-conjugated anti-CD45 all at 1:200 for 15 min at 4 ºC. Neutrophils were identified as CD45^+^CD11b^+^Ly6G^+^, and numbers were estimated with PEB/DAPI containing True Count Beads (BD Biosciences).

### Quantification of neutrophil numbers in cryopreserved infarcted hearts and skin wounds

Infarcted mice were anesthetized and euthanized as described above (AMI model). Then, 2.5 mL of PBS was infused intravenously through the inferior vena cava to remove blood and the heart was extracted. The infarcted heart was minced and digested in HBSS with liberase (1U/ml, Roche) and DNAse I (10mU/ml, Sigma) for 45 min at 37°C. Then, we obtained a single-cell suspension by gentle pipetting and mechanical dissociation of the remaining pieces through 70 μm-cell strainers (BD Falcon). We lysed the samples with RBC 1x Lysis Buffer for 5 minutes at RT and washed them with PBS. Finally, the single-cell suspension obtained was resuspended in 1 mL of FBS with 20% DMSO, put in cryotubes and saved within Cryo Freezing Containers (Mr. Frosty) at -80 ºC. On the day of analysis, all samples were thawed for 1 minute at 37 ºC and immediately placed on ice. A fraction of each sample was taken, washed twice with PEB to remove the FBS and stained with AF647-conjugated anti-Ly6G (1:200), APC Cy7-conjugated anti-CD45 (1:200) and FITC-conjugated anti-CD11b (1:200) for 15 min at 4 ºC. Total numbers of neutrophils were estimated with PEB/DAPI containing True Count Beads (BD Biosciences). To quantify neutrophils in ear skin wounds, we performed the wound model described above and harvested the ear skin 24 hours after injury. We then minced and digested the skin in HBSS with liberase (1U/ml, Roche) and DNAse I (10mU/ml, Sigma) for 1 hour at 37°C. Single-cell suspensions were obtained by gentle pipetting and mechanical dissociation of the remaining pieces through 100 μm-cell strainers (BD Falcon). The single-cell suspension was lysed with RBC 1x Lysis Buffer for 5 minutes at RT, washed with PEB and stained with AF647-conjugated anti-Ly6G (1:200), APC Cy7-conjugated anti-CD45 (1:200) and FITC-conjugated anti-CD11b (1:200) for 15 min at 4 ºC. Finally, the total numbers of neutrophils were estimated with PEB/DAPI containing True Count Beads (BD Biosciences).

### Quantification of leukocytes in infarcted mouse hearts

Infarcted mice (control and neutrophil-depleted) were anesthetized and euthanized as described above (AMI model). We infused 2.5 mL of saline intravenously through the inferior vena cava to remove blood and the heart was extracted. The infarcted heart was minced and digested in HBSS with liberase (1U/ml, Roche) and DNAse I (10mU/ml, Sigma) for 45 min at 37°C. Then, we obtained a single-cell suspension by gentle pipetting and mechanical dissociation of the remaining pieces through 70 μm-cell strainers (BD Falcon). We lysed the samples with RBC 1x Lysis Buffer for 5 minutes at RT and washed them with PBS. Finally, the single-cell suspension obtained was processed for FACS analysis. A fraction of each sample was taken, washed with PEB and stained with PerCPCy5.5-conjugated anti-CD45 (1:200), BV510-conjugated anti-CD11b (1:200), BUV737-conjugated anti-Ly6G (1:200), BV711-conjugated anti-Ly6C (1:100), BV650-conjugated anti-CD64 (1:50), FITC-conjugated anti-CD45R (1:200), PE Cy7-conjugated anti-CD4 (1:200), APC Cy7-conjugated anti-CD8a (1:200), PE-conjugated anti-CCR2 (1:50) and APC-conjugated anti-SiglecF (1:200) for 15 min at 4 ºC. The total numbers of each immune cell population were estimated with PEB/DAPI containing True Count Beads (BD Biosciences).

### Zymosan-induced peritonitis

We treated mice with zymosan (1mg, intraperitoneal injection, Sigma). After 2h, we took blood samples and performed peritoneal lavage with 10 ml of cold PBS for cytometric analyses and cell counts. Briefly, a fraction of blood samples and peritoneal lavages were stained with AF647-conjugated anti-Ly6G (1:200) or DyLight 450-conjugated anti-Ly6G (1:200) for 15 min at 4 ºC. The rest of blood was analysed in automated hemocytometers (Abacus Junior, Diatron; ABX Pentra 80, Horiba). In bone marrow-transplanted mice, we compared the ratios of neutrophils in the peritoneum and blood to estimate the migration efficiencies of mutant (DAPI^NEG^Ly6G^+^DsRed^NEG^) and WT (DAPI^NEG^Ly6G^+^DsRed^+^) cells (ratio in peritoneum/ratio in blood). In non-chimeric mice, we compared the migration efficiency of neutrophils in control and agonist-treated mice measuring absolute number of neutrophils (DAPI^NEG^Ly6G^+^) in the peritoneal lavage using PEB/DAPI containing True Count Beads (BD Biosciences). We normalized migration relative to the absolute number of neutrophils in blood.

### ROS and intracellular IL-1β

Red blood cell-lysed blood and peritoneal lavage were plated in RPMI in 96-well polystyrene microplates (Corning Falcon) and stimulated with 133 nM of phorbol 12-myristate 13-acetate (PMA) (Sigma) for 20 min at 37 ºC or with RPMI alone for basal condition. Cells were then stained with AF647-conjugated anti-Ly6G (or FITC-conjugated anti-Ly6G for posterior IL-1β staining) and PE-conjugated anti-CD11b for 15 min at 4 °C. For ROS measurement, cells were additionally stained with 5mM DHR123 (Invitrogen) for 20 min at 37 °C. Geometric Mean Fluorescence Intensity (GMFI) of DHR123 in neutrophils (DAPI^NEG^CD11b^+^Ly6G^+^) was obtained to measure ROS production. For detection of IL-1β production, after staining for cytometric analysis, cells were fixed and permeabilized using the Fix/Perm and Perm Buffers (eBiosciences) according to manufacturer’s instructions. Cells were then stained with APC-conjugated anti-IL1β for 15 min at 4 °C and we calculated the median fluorescence intensity (MFI) of IL-1β levels in neutrophils (DAPI^NEG^CD11b^+^Ly6G^+^).

### CXCR2 and CXCR4 cross-inhibition and CXCR4 internalization assays

Neutrophils were purified from BM of wild-type mice with Percoll (GE Healthcare). RBCs were lysed, neutrophils resuspended in RPMI 1640 (Invitrogen) with FBS and antibiotics and left incubated overnight at 37 ºC with 5% CO_2_. Some cells were pretreated with CXCL12 (10 nM, R&D Systems) or ATI-2341 (3 μM, Tocris) for 5 minutes at 37ºC, whereas others were left untreated. Cells were allowed to migrate towards CXCL1 (50 nM, R&D Systems) through 6.5 mm transwells with 5μm pore polycarbonate membrane insert (Corning) for 1 hour at 37 ºC, 5% CO_2_. Transmigrated cells were collected, washed with 1 ml of PEB and stained with AF647-conjugated anti-Ly6G for 15 min at 4 ºC for cytometric analysis. We then measured the number of transmigrated neutrophils (DAPI^NEG^Ly6G^+^) using PEB/DAPI containing True Count Beads (BD Biosciences) and used migration to only media as a control. For evaluation of CXCR4 internalization, cells were pretreated with CXCL12 (10nM, R&D Systems) or ATI-2341 (from 30nM to 30 μM, Tocris) for 60 minutes at 37ºC while others were left untreated. Cells were collected and stained with AF647-conjugated anti-Ly6G, PE-conjugated anti-CXCR4 and PerCPCy5.5-conjugated anti-CXCR2 for 15 min at 4 ºC for cytometric analysis. Median Fluorescence Intensity (MFI) was obtained for CXCR4 and CXCR2 markers gated on live neutrophils (DAPI^NEG^Ly6G^+^).

### Culture and analysis of neutrophils

Bone marrow and blood neutrophils from wild-type and mutant mice were cultured in RPMI 1640 (Invitrogen) with FBS at 37 ºC with 5% CO_2_ for 6h to simulate the natural aging process, in the absence and/or presence of CXCL12 (5nM for blood and 10nM for BM, R&D Systems). We collected samples at different times (0, 2, 4 and 6h) for cytometric staining by incubating the cells with AF647-conjugated anti-Ly6G (1:200), FITC-conjugated anti-CD62L (1:200), PerCP-Cy5.5-conjugated anti-CXCR2 (1:200), PE-conjugated anti-CXCR4 (1:200), BV510-conjugated anti-CD11b (1:200) and PE Cy7-conjugated anti-CD101 (1:200). Median Fluorescence Intensity (MFI) was obtained for those markers gated on alive neutrophils in blood (DAPI^NEG^CD11b^+^Ly6G^+^) and alive mature neutrophils in BM (DAPI^NEG^CD11b^+^Ly6G^+^ CD101^+^).

### Intravital microscopy (IVM) of the cremaster muscle (2D and 4D)

To evaluate neutrophil behaviour within the microvasculature during inflammation, intravital microscopy of the cremaster muscle after TNFα stimulation (R&D Systems, 0.5μg intrascrotal injection) was performed as previously reported (Adrover et al., 2019; Hidalgo et al., 2009) using the VIVO system (Intelligent Imaging Innovations, Denver, CO). We used a plan-Apochromat 40x W NA1.0 ∞/0 objective (Zeiss) and the SlideBook software (Intelligent Imaging Innovations) for image acquisition. For the 2D motility analysis of mutant, treated or control neutrophils, 6-12 venules per mouse were analyzed 150 to 210 min after TNF-α treatment by epifluorescent imaging acquisition (Cy3/561 channels for PE, FITC/488 channels for FITC and Cy5/640 channels for APC) and bright-field images with 2×2 binning with a 3 s interval for 2 min on each field of view. For the visualization of leukocytes, mice were injected intravenously with 1μg of fluorescently labeled anti-Ly6G-APC or anti-Ly6G-FITC, and 1 µg of anti-CD41-PE to visualize platelets. For 4D intravital imaging, 6-12 venules per reporter mouse were analyzed 150 to 210 min after TNF-α treatment using laser stacks for 488, 561 and 640nm beams coupled with a confocal scanner (Yokogawa CSUX-A1; Yokogawa). Full Z stacks that covered a similar cylindrical segment of the venules with an average depth of 26µm with 2µm Z-intervals were acquired for approximately 6 minutes. For visualization of the vessel wall, mice were injected with 1µg of fluorescently labeled anti-CD31-APC in combination with TNF-α as published (Goh et al., 2018).

### Behavioural analysis of intravascular neutrophils

For analysis of rolling and adhered cells to the inflamed endothelium, we used the ImageJ software (NIH). Rolling neutrophils along venules within one minute were quantified and adhered neutrophils were quantified as those remaining stationary for at least 1 min. Counts of rolling or adhered cells in 2-minute captures (captured at 3 second intervals) were normalized using the width of the vessel to allow comparison among all vessels. For adhesion or rolling efficiency indices, this data was compared with the frequency or numbers of free-flowing WT and experimental cells in the blood for each mouse, which was obtained from cytometric analysis of blood neutrophils for BM chimeric mouse or from haematological analysis for experimental mice. For 4D intravital analyses, we assessed the behaviour of intravascular neutrophils using ACME (Automated Cell Migration Examination), as previously described (Crainiciuc et al., 2022; Molina-Moreno et al., 2022). Briefly, ACME is an automatic feature extraction method for cell migration analysis in microscopy imaging that combines deep learning and machine learning blocks to segment, track and extract features from cells moving along blood vessels. The raw data generated by ACME was uploaded to RStudio and the input parameter expression matrix had 73 parameters for X cell points (objects), corresponding to X unique cells, including metadata information. We used the Seurat v4 package functions to pre-process and analyze the data. First, we transformed the data matrix to a Seurat Object and the parameters were scaled. Normalization for each cell was not necessary. We performed Principal Component Analysis (PCA) to reduce the dimensionality to the top principal components and generated UMAP plots of the dataset. For unsupervised clustering, we performed k-means algorithm over the data previously mentioned. With the unbiased labelling per cell obtained, we added this brand-new information to the Seurat object previously created. This allowed us to see the value distribution of each morpho-kinetic parameter for each cell/group, and according to the patterns, we classified these unbiased groups with the canonical behavioral label classification (Crainiciuc et al., 2022), in accordance with their parameter traits.

### Intravital microscopy of the cremaster muscle in SCD mice

To analyze the function of daytime, nighttime and CXCR4-activated neutrophils during vascular injury, ATI-treated or control male SCD mice were administered with 0.5µg TNF (R&D Systems) intraperitoneally and anesthetized two hours later by a mixture of 150mg/kg α-chloralose (Sigma-Aldrich) and 1.2g/kg urethane (Sigma-Aldrich) in PBS. Tracheal intubation was performed to ensure normal and spontaneous respiration after anesthesia. The cremaster muscle was gently exteriorized, mounted onto a microscopic stage, and continuously superfused with bicarbonate-buffered saline (Ringer’s solution, pH 7.4, 37 °C). A minimum of 3 up to 10 postcapillary venules (15-25μm of diameter) per mouse were visualized for 30-45 minutes using a custom-designed upright microscope equipped with a 60X water immersion objective. Each venular segment (100mm) was recorded for one minute using a video camera containing a charged-coupled device (CCD camera, Hamamatsu). For analysis of rolling, adhesion and cell interactions in SCD mice, images were recorded on a Sony SVO-9500MD videocassette recorder (Sony), digitalized using a Dazzle DVC 170 Recorder HD (Corel Corporation) and processed using the Pinnacle Video Creator software 1.0.1 (Corel Corporation). During image acquisition, rolling cells along venules within one minute were quantified. Adherent cells were quantified as the number of leukocytes remaining stationary for one minute. Interactions between sRBC and WBC were defined as the associations between a sRBC and an adherent or rolling leukocyte for more than one second. The centerline RBC velocity (V_RBC_) for each recorded venule was measured using an optical Doppler velocimeter (Texas A&M). Blood flow rate (Q) was calculated as *Q = V*_*mean*_ *x πd*^*2*^*/4* where *d* is the venule diameter and *V*_*mean*_ is the centerline velocity, estimated as V_RBC_ / 1.6. Survival times were defined as the time from TNFα injection until death, addressed up to 360 minutes.

### Whole-mount immunostaining and tissue clearing

For imaging of different tissues, these were extracted and fixed in PFA 4% overnight after euthanizing and perfusing mice with 10 ml of PBS. Then, tissue samples were permeabilized in methanol gradients in PBS for 30 min (PBS > MetOH 50% > MetOH 80% > MetOH 100%), bleached with Dent’s bleach (15% H_2_O_2_, 16.7% DMSO in MetOH) for 1h at RT and rehydrated through descending methanol gradients in PBS (MetOH 80% > MetOH 50% > PBS). We incubated the samples with blocking buffer containing PBS with 0.3% Triton X100, 0.2% BSA, 5% DMSO, 0.1% azide and 25% FBS overnight at 4ºC with shaking. Afterwards, we stained the samples with primary antibodies for 2 days at 4ºC with shaking. After washing for 24h in washing buffer (PBS with 0.2% Triton X100 and 3% NaCl), tissues were stained with secondary antibodies for 2 days at 4ºC with shaking and later washed in washing buffer for 24h. Before imaging, tissues were dehydrated in MetOH gradients in dH_2_0 using glass containers for 30 min in each step (MetOH 50% > MetOH 70% > MetOH 90% > 3x MetOH 100%), cleared for 30 min in MetOH with 50% BABB and afterwards in 100% BABB (benzyl alcohol, benzyl benzoate 1:2) and imaged at the Advanced Microscopy unit at CNIC.

### Immunofluorescence of infarcted hearts

For identification of neutrophils in the damaged myocardium, infarcted mice were injected intravenously with 10µg of AF647-conjugated anti-VeCadherin (clone BV13 Biolegend) 10 minutes before sacrifice for identification of blood vessels and infarcted area. Then, mice were perfused with 1 ml of PBS, and the infarcted heart was extracted and fixed in PFA 4% overnight. After washing with PBS, we processed the tissue as indicated above for tissue clearing. Infarcted hearts were stained with biotinylated anti-myeloperoxidase (MPO, R&D Systems) as primary antibody at 1:200 for detection of neutrophils. As secondary antibodies, we used goat Alexa-488 conjugated Streptavidin (Biolegend) at 1:400 and DAPI (Life Technologies) at 1:1000 during the last hour of staining. Finally, we imaged the hearts in a Leica SP8 X confocal microscopy system coupled to a DMI6000 inverted microscope with 40x/1.3 oil magnification taking tile-scan images with around 150-200µm Z-stack at the Advanced Microscopy unit at CNIC. Neutrophils within border zones (VeCadh+; close to the infarcted area and no more than 150 µm close to the edge of the heart) or infarcted areas (VeCadh^NEG^; ischemic area) were quantified as MPO+ cells with Imaris (Bitplane) by applying the spots model for quantification in 1µm^3^ regions.

### Immunofluorescence of the wounded ear skin

To identify neutrophils in the wounded ear skin, we performed wounds in the ear skin as described above and harvested them after injury. We fixed them in PFA 4% overnight, washed with PBS and hair was removed by the application of depilatory cream for 10min (Veet). Ears were washed again in PBS for 1h and wounded regions were isolated from the whole tissue (approximately 3.0 mm by 1.5 mm) without removing the cartilage. Samples were processed as indicated above for tissue clearing. The samples were stained with biotinylated anti-MPO (R&D Systems), anti-RFP (Rockland) or anti-Mrp14 (Abcam), and anti-CD31 (Thermo Fisher) as primary antibodies at 1:200 for detection of neutrophils and vessels, respectively. As secondary antibodies, we used Alexa-488 conjugated Streptavidin (Biolegend), goat anti-rabbit-Alexa 568 (Life Technologies) or goat anti-rat-Alexa 488 (for neutrophil identification) and goat anti-hamster-Alexa 647 (Life Technologies) (for vessel identification) at 1:400. DAPI (Life Technologies) was also added at 1:1000 during the last hour of staining. Finally, we imaged the wounds in a Leica SP5 multiline inverted confocal microscope or a Leica SP8 X confocal microscopy system coupled to a DMI6000 inverted microscope with 20x/0.7-0.75 imm (oil) or 40x/1.25-1.3 oil magnification taking 4x4 tile-scan images with whole-section Z-stack at the Advanced Microscopy unit at CNIC. We identified wounded areas as those surrounded massively by cells (DAPI+) and neutrophils (MPO+, RFP+ or Mrp14+), forming a cauterizing ring. We quantified MPO+, RFP+ or Mrp14+ cells in a 1000x1000µm area to estimate the number of neutrophils around the wound (center of the image) and examined their distribution using Imaris (Bitplane) by applying the spots and surface model for quantification of neutrophils.

### Multiphoton imaging of the ear skin for second harmonic generation (SHG)

Fixed ear wounds obtained as previously described were imaged with a confocal-multiphoton microscope (Zeiss LSM 780 Upright) with a 20X/1.0 water objective. Excitation wavelength used was 850 nm (Laser Coherent Chameleon Vision-S). Bandpass filters used were 445/50 (SHG) and 525/50 (GFP), along with brightfield, taking 2x2 tile-scan images with Z-stack of approximately 60-100µm. Wound size was determined based on SHG signal and analysis was performed using ImageJ software (NIH) by directly delimiting area.

### In situ apoptosis detection (TUNEL assay)

For quantification of apoptotic areas in the wounded ear skin we used a commercially available kit for TUNEL assay (Thermo Fisher) and followed the manufacturer’s protocol using p96 plates for embedding the wounded regions that were obtained following the previously described ear wound model in Ly6G^TdTom^ mice. Then, samples were processed as indicated above for tissue clearing, using additional anti-RFP (Rockland, 1:200) and goat anti-rabbit-Alexa 568 (Life Technologies, 1:400) antibodies for neutrophil identification, and DAPI (Life Technologies, 1:1000). Imaging was performed using a Leica SP5 multiline inverted confocal microscope or a Leica SP8 X confocal microscopy system coupled to a DMI6000 inverted microscope with 40x magnification taking 3x3 tile-scan images with whole-section Z-stack at the Advanced Microscopy unit at CNIC. TUNEL signal was quantified with ImageJ software (NIH).

### Annexin V staining in wounds

24 h after skin injury ears were harvested and placed on cold excision buffer (PBS 1x, 10% FBS). Hair was removed by the application of depilatory cream for 5 min (Veet) and washed with excision buffer. Then, ears were incubated without light, shaking and at 4 ºC for 15 min in a Annexin V binding buffer 1x (dH_2_O, 10mM Hepes, pH 7.4, 140mM NaCl, 2.5mM CaCl_2_) with anti-Annexin V PE-conjugated (BD Biosciences) at 1:200 and DAPI (Life Technologies) at 1:1000. We washed again the samples with Annexin V binding buffer and 3-4 wounds per ear were imaged in a Leica SP5 multi-line inverted confocal microscope with 20x magnification. Annexin V signal around the wound was identified and intensity and area analyzed using ImageJ software (NIH).

### *Ex vivo* NET-formation assay

DAPI^NEG^Ly6G^+^ blood neutrophils were sorted and 5x10^4^ neutrophils were plated with RPMI 1640 (Invitrogen) medium on Poly-L-lysine covered 8-well µ-slides (Ibidi) and left 30 min to adhere in the incubator at 37 ºC, 5% CO_2_. Cells were then incubated in the same conditions for 2 h with 100 nM PMA (Sigma) or RPMI as vehicle. Neutrophils were fixed using PFA 4% for 10 min and washed with PBS. Cells were then permeabilized with 5% BSA, 1% normal goat serum, 5% FBS and 0.1% triton-x 100 in PBS for 30 min. Staining of neutrophil extracellular traps was performed using antibodies against citrullinated histone 3 (cit-H3, Abcam) and biotinylated-MPO (R&D Systems) at 1:200 in PBS for 3 hours at RT. After washing with PBS, cells were incubated with secondary antibody goat anti-rabbit-Alexa 568 (Life Technologies) and Alexa-647 conjugated Streptavidin (Life Technologies) at 1:400 in PBS for 1.5 hours at RT. Cells were also counterstained with SYTOX Green (Invitrogen) to reveal free DNA and nuclei. Finally, samples were washed with PBS and Mowiol 4-88 (Mw 31,000, Sigma) was added as mounting medium. Imaging of NETs was performed using a Leica SP5 multi-line inverted confocal microscope with 40x/1.25 oil magnification, taking 5x5 tile-scan images with whole-slide Z-stack. We analyzed the images using Imaris (Bitplane) and identified NETs by the triple colocalization of the DNA, MPO and cit-H3 channels, by applying the spots model for quantification in this new channel.

### Single-cell RNA sequencing of heart cells (scRNA-seq)

For single cell analysis of heart cell populations, control and infarcted mice (24h of reperfusion) were euthanized with CO_2_, and the heart was collected after perfusion with 5ml of PBS. Hearts were minced and digested in HBSS with liberase (1U/ml, Roche) and DNAse I (10mU/ml, Sigma) for 45 min at 37°C. Then, we obtained a single-cell suspension by gentle pipetting and mechanical dissociation of the remaining pieces through 70 μm-cell strainers (BD Falcon). We lysed the samples with RBC 1x Lysis Buffer for 5 minutes at RT and washed them with PBS. Finally, samples were stained for sorting. Single-cell suspensions were sorted in a BD FACS Aria II SORP Cell Sorter and a BD FACSAria Fusion Cell Sorter as DAPI^NEG^CD45^+^, DAPI^NEG^CD45^-^CD31^+^ and DAPI^NEG^CD45^-^CD31^-^PDGFRα^+^ cells, and loaded into a BD Rhapsody cartridge. For the generation of single-cell whole-transcriptomes we used a BD Rhapsody system according to manufacture instructions. A pool of 12 samples of labelled Sample Tags cells was checked for viability and cell concentration using the Countess III cell counter (Thermofisher). Up to 60.000 cells were loaded into a Rhapsody Single Cell Analysis Systemcartridge. Cell capture and cDNA synthesis were performed according to manufacturer’s instructions. Basically, cells are isolated into nanowells by gravity, then cells are lysed and mRNA and Sample tags oligos are released and captured by beads present in the nanowells. Each bead contains a unique oligo named “cell label” to identify each individual bead. All beads present in the cartridge are collected and cDNA synthesis takes place in a single reaction. At this point each cDNA and Sample Tag oligo is attached to its corresponding “cell label” oligo. Two separated indexed libraries were prepared for whole transcriptome analysis and Sample Tag demultiplexing following manufacturer’s instructions. The average size of the libraries was calculated using the 2100 Bioanalyzer (Agilent) and the concentration was determined using the Qubit® fluorometer (Thermofisher). Finally, libraries were combined and sequenced together in a paired end run (51x75) using a NextSeq 2000 system (Illumina) and a P2 flowcell. Output files were processed with NextSeq 1000/2000 Control Software Suite v1.4.1. FastQ files for each sample were obtained using BCL Convert v3.6.3 software (Illumina).

Single cell raw FastQ files were processed using BD’s Rhapsody v2.2 pipeline and aligned to the mm10 mouse reference (RhapRef_Mouse_WTA_2023-02.tar.gz) to obtain gene matrix counts. This pipeline includes steps for quantification and filtering of low-quality cells and tagging of doublets, which were also filtered out of the downstream analyses. All downstream analyses were performed using Seurat v4.3.0.1 on R version 4.0.3. (Hao et al., 2021).

### Chemokine quantification in plasma

CXCL12 amount was measured in plasma samples taken every 4h from WT mice using commercially available ELISA reagents, following the manufacturer’s protocol (R&D Systems). Plasma samples were obtained by two sequential centrifugations of total blood obtained by cardiac puncture at 10000g for 15 min and 1000g for 10 min, respectively.

### Western Blotting

Neutrophils were purified from BM of wild-type mice with Percoll (GE Healthcare). RBCs were lysed, neutrophils resuspended in RPMI 1640 (Invitrogen) with FBS plus antibiotics and left incubated overnight at 37ºC with 5% CO_2_. An aliquot of the cells was pretreated with CXCL12 (50nM, R&D Systems) or ATI-2341 (3µM, Tocris) for the indicated times at 37ºC. Cells were lysed in RIPA buffer containing 50 mM Tris-HCl, pH 8; 150mMNaCl; 1% Triton X-100; 0.5% sodium deoxycholate; 0.1% SDS; 1mM PMSF (Sigma) and a protease inhibitor cocktail (Sigma). Proteins from 1.5x10^5^ lysed cells were separated by 10% SDS-PAGE and transferred onto PVDF membrane. Membranes were incubated overnight with antibodies against p-pERK1/2 (Cell Signaling) and pERK1/2 (Cell Signaling) at 1:500 dilutions, and then thoroughly washed and incubated with secondary antibody goat anti-rabbit-Alexa 647 (Life Technologies) at 1:5000 dilution. Blots were visualized using the imaging system iBright 1500 and blots quantified with ImageJ.

### Human data analysis

The clinical study was a retrospective analysis of the administrative and clinical data from patients hospitalized with ST-segment elevation myocardial infarction (STEMI) diagnosis as coded in the discharge report (International Classification of Diseases, 9th revision, clinical modification codes 410.0-410.6 and 410.8 until 2015, and International Classification of Diseases, 10th Revision, Clinical Modification codes I21.0-I21.4, I22.0, I22.1, I22.8 and I22.9 since 2016) at Hospital Universitario 12 de Octubre, a large volume tertiary centre in Madrid (Spain). The data was collected between January 1, 2010 and December 31, 2021. We excluded cases with a) age<18 years, b) absence of ST-elevation in the initial electrocardiogram, c) presence of non-obstructive coronary disease in the initial coronary angiography, d) presence of diagnostic coding errors in hospital records, e) absence of matching with the acute coronary syndrome registry of the Cardiology Department or f) absence of blood test analysis during admission.

Patient baseline characteristics, Killip class, reperfusion and standard times (including time from symptom onset, first electrocardiogram, time of primary angioplasty and total ischemic time) were recorded. Information from coronary angiographies was also collected, including location of coronary artery occlusion using the Bypass Angioplasty Revascularization Investigation classification system, result of the procedure (procedural success, including Thrombolysis In Myocardial Infarction flow grade). Left ventricular damage assessed by the left ventricular ejection fraction measured by 2-dimensional echocardiography during admission and classified as normal (50-70%), mild dysfunction (40-49%), moderate dysfunction (30-39%), and severe dysfunction (less than 30%) and by peak level of cardiac troponin T during serial measurement. Other blood test parameters included haemoglobin, platelet count, leukocyte count, and neutrophil count. The database was pseudonymized and data analysed anonymously so no informed consent was required. The study was approved by the investigation review board (Clinical Research Ethics Committee) of the Hospital Universitario 12 de Octubre (Project: Study of the circadian rhythm in neutrophils and its influence on acute myocardial infarction).

### Statistical analysis

Unless otherwise indicated, all data are shown as the mean ± standard error of the mean (SEM). For comparisons between two groups we applied Student’s paired or unpaired t-test. COSINOR fitting of circadian curves was performed to determine diurnal patterns, using the curve-fitting module of GraphPad Prism with the equation *Y = Baseline + Amplitude x cos (Frequency X + Phaseshift)*, where Baseline = average of Ymax and Ymin, Amplitude = 0.5 x (Ymax – Ymin), Frequency = 0.2618 (2π/24) and Phaseshift = value of X at Ymax. The oscillating pattern was determined by comparing the COSINOR-calculated amplitudes with a hypothetical zero-amplitude curve assuming identical standard deviations and using unpaired t-test analyses, as previously described (Adrover et al., 2019). For data with more than two data sets, we used one-way analysis of variance (ANOVA) with Turkey’s or Dunnett’s multigroup tests. Comparisons of two-time curves were performed using two-way ANOVA with Šídák multigroup test. The log-rank Mantel-Cox test was employed to determine statistical differences between Kaplan-Meier survival curves. Sample exclusion was not performed unless evident signs of disease were found in a mouse, in which case statistically significant outliers were identified using Grubb’s test (ESD method). Analyses were performed with the GraphPad Prism v9 software (GraphPad). P values <0.05 were considered statistically significant; non-significant differences (n.s.) are indicated accordingly.

## Supplementary Material

Supplementary Material

## Figures and Tables

**Table 1:** Baseline characteristics and clinical data of STEMI patients. The original data was collected from 2043 patients for the demographic study. 2 patients were outliers for neutrophil counts, and the other 15 were excluded for code and data mismatch. The table shows the relevant information for the 2026 patients included in the analyses shown in Figure 1.

	Total	Male	Female
n	2043	1569	474
Male %	76.8%	-	-
Female %	23.2%	-	-
Age (years)	69.2 ± 13.7	67.5 ± 13.1	75.0 ± 14.0
Weight (kg)	80.8 ± 45.8	84.2 ± 51.1	69.7 ± 15.5
Height (m)	1.68 ± 0.36	1.71 ± 0.37	1.59 ± 0.34
Max. troponin	4985 ± 5959	5067 ± 5868	4710 ±6249
FEVI I (n)	767	573	194
FEVI II (n)	418	328	90
FEVI III (n)	196	156	40
FEVI IV (n)	56	41	15
FEVI missing (n)	606	471	135
Ischemia (%)	4.43 ± 3.81	4.33 ± 3.83	4.76 ± 3.72
Neutrophils x10^3^/μl	9.58 ± 3.96	9.65 ± 4.01	9.37 ± 3.78
Leukocytes x10^3^/μl	12.2 ± 4.74	12.3 ± 4.11	12.1 ± 6.4
Lymphocytes x10^3^/μl	1.79 ± 2.57	1.73 ± 1.06	1.98 ± 4.96

## Data Availability

All data generated in this study are presented in the manuscript and/or supplementary material. Any further information required for replicating experimental procedures will be made available by the corresponding authors upon reasonable request. Transcriptomic data underlying Fig. S3 are available in Gene Expression Omnibus (GEO). Individual GEO accession number: GSE304501 (GSM9151587 and GSM9151588).

## Non-standard abbreviations

AAR: Area At Risk
ACME: Automated Cell Migration Examination
AMI: Acute Myocardial Infarction
BM: Bone Marrow
Cit-H3: Citrullinated Histone 3
CVD: Cardiovascular Diseases
DHR123: Dihydrorhodamine 123
DT: Difteria Toxin
EF: Ejection Fraction
FS: Fraction Shortening
GEO: Gene Expression Omnibus
I/R: Ischemia/Reperfusion
IVM: Intravital Microscopy
IVS: Intraventricular Septum
LA: Left Atrium
LAD: Left Anterior Descending
LV: Left Ventricule
LVPW: Left Ventricule Posterior Wall
MFI: Median Fluorescence Intensity
MPO: Myeloperoxidase
MRI: Magnetic Resonance Imaging
NETs: Neutrophil Extraceullar Traps
PFA: Paraformaldehyde
RBC: Red Blood Cell
SCD: Sickle Cell Disease
scRNAseq: Single Cell RNA Sequencing
SHG: Second Harmonic Generation
sRBC: Sickle Red Blood Cell
STEMI: ST-Segment Elevation Myocardial Infarction
tSNE: t-distributed Stochastic Neighbour Embedding
TUNEL: Terminal deoxynucleotidyl transferase dUTP Nick-End Labeling
UMAP: Uniform Manifold Approximation and Projection
WBC: White Blood Cell
ZT: Zeitgeber
