# Supplementary material for: A circadian checkpoint relocates neutrophils to minimize injury
Source: J Exp Med. Author manuscript; Available in PMC 2026 Feb 2. (PMC7618653; doi:10.1084/jem.20250240)
Supplement: Supplementary Material [file EMS212082-supplement-Supplementary_Material.docx]

**Supplemental Material**

Supplementary Figures 1 to 5. Fig. S1 shows the effect of neutrophil depletion at tissue level in homeostasis and after myocardial infarction. Fig. S2 provides additional data describing the circadian activity and number of cells in circulation of neutrophil-depleted, CXCR4, and Bmal1 mutant mice. Fig. S3 presents the transcriptional changes that occur in the different heart cell populations after treatment with the CXCR4 agonist ATI2341 during myocardial infarction. Fig. S4 shows the effect of the CXCR4 agonist ATI2341on vascular permeability. Fig. S5 presents additional data showing the distribution of neutrophils in injured tissues.

**Supplementary Fig.1. Depletion of neutrophils in myocardial infarction experiments.** (a) Number of neutrophils in circulation in control and neutrophil-depleted mice after bleeding mice at different circadian times; n=2 mice per time point. The diurnal curves are repeated to appreciate the circadian pattern better. (b) Gating strategy for neutrophil identification after depletion. (c) Experimental scheme for the ischemia-reperfusion (AMI) model at two different circadian times and antibody cocktails used as control or to deplete neutrophils. ZT indicates the time of infarct. Analysis was performed 1h and 45min later. (d) Areas at risk of the myocardium (left) in control and neutropenic mice at ZT5 or ZT13 and infarct sizes (right) after correction for areas at risk from the same animals subjected to MI and followed by one hour of reperfusion; n=4 mice per group. (e) Experimental scheme for evaluation of immune cell populations in the blood and heart of naïve and infarcted mice after neutrophil-depletion. Analysis was performed 24h after reperfusion. (f) Heatmap representing the numbers of the indicated immune cell populations in the blood of control and neutrophil-depleted mice at ZT5, in basal conditions and after AMI; n=4 mice per group (for naïve) and n=3-4 mice per group (for AMI). Data are normalized to the control isotype (iso) group for each cell type. (g) Heatmap representing the numbers of the indicated immune cell populations in the heart of control and neutrophil-depleted mice at ZT5, in basal conditions and after AMI; n=4 mice per group (for naïve) and n=3-4 mice per group (for AMI). Data are normalized to the control isotype (iso) group for each cell type. Data in (a-d) are from single experiments. Data in (f and g) are pooled from two experiments. Data are shown as mean ± SEM. *p<0.05; **p<0.01; ***p<0.001; ns, not significant, as determined by amplitude vs. zero test (a), two-way ANOVA (d) and unpaired t-test (f, g).

**Supplementary Fig.2. Circadian activity and blood counts of neutrophil-depleted, CXCR4, and Bmal1 mutant mice.** (a) Myeloid cells and neutrophils at the indicated circadian times in the blood of WT, Bmal1^ΔN^, Cxcr4^+/1013^ mice; n=3-4 mice per group. (b) Peripheral blood counts in WT littermates, Cxcr4^+/1013^ (WHIM) (up) and Bmal1^ΔN^ (bottom) mice at ZT5; n=7-9 mice per group for Cxcr4^+/1013^ and n=5 mice per group for Bmal1^ΔN^. (c) O_2_ consumption (VO2), CO_2_ production (VCO2), energy expenditure (EE), respiratory quotient (RQ), general locomotor activity (activity), vertical activity (rearing), food intake (food) and drink intake (drink) of wild-type, Bmal1^ΔN^, Cxcr4^+/1013^ and neutrophil-depleted mice housed in metabolic cages for 3 days with food and water available ad libitum; n=3-14 mice per group. AUs: Arbitrary Units. (d) Experimental scheme for evaluation of cardiac function by MRI in wild-type and Bmal1^ΔN^ after performing AMI (45’ occlusion; 24h reperfusion) at the indicated ZTs. (e) Ejection fraction measured by MRI at day 0 and day 7 in wild-type and Bmal1^ΔN^ mice subjected to AMI and followed for 24 hours of reperfusion at the indicated ZTs; n=3-4 mice per group. (f) Difference in ejection fraction (ΔEF) between day 0 and day 7 in mice from (e); n=3-4 mice per group. Data in (a and c-f) are from single experiments. Data in (b (bottom; Cxcr4^+/1013^) are pooled from two experiments. Data in (b (up; Bmal1^ΔN^) are representative from two experiments. Data are shown as mean ± SEM. *p<0.05; **p<0.01; ***p<0.001; ns, not significant, as determined by unpaired t-test analysis (b, f) and two-way ANOVA (a, c, e).

**Supplementary Fig.3. Transcriptional profile of heart cell populations after CXCR4 activation during AMI.** (a) Experimental scheme. Indicated cell populations were sorted from control and infarcted hearts from ATI2341- and vehicle-treated mice. (b, c) UMAP visualization of the scRNAseq analysis of total cells coloured by group of origin (b) and treatment (c). (d) UMAP visualization of the scRNAseq analysis of total cells coloured by type of cell (C0-C6) and top 8 genes expressed by each cluster. (e, f) Venn diagrams of detected genes in macrophages and neutrophils (e), and endothelial cells and fibroblasts (f) in control and AMI groups. (g, h) Volcano plot representing DEG from detected genes in macrophages and neutrophils (g), and endothelial cells and fibroblasts (h) during AMI. Data in (a-h) are from a single experiment.

**Supplementary Fig.4. ATI2341 does not protect from vascular leakiness.** (a, b) Vascular leakiness in SCD administered with vehicle or ATI2341without (left) and with TNFα administration (right); n=4 mice per group. (c) Vascular leakiness in vehicle- and ATI2341-treated mice in basal conditions (left) and after LPS challenge (right); n=4 mice per group. (d) Vascular leakiness in vehicle- and ATI2341-treated mice in basal conditions and after induction of AMI; n=3-4 mice per group. Data in (a and b) are from a single experiment. Data in (c and d) are pooled from two experiments. Data are shown as mean ± SEM. ns, not significant, as determined by two-way ANOVA (a, c and d) and unpaired t test (b).

**Supplementary Fig.5. Redistribution of neutrophils around wounds.** (a) Experimental scheme of procedure for wound healing model in the ear. (b) Representative image for neutrophil recruitment to the ear wound 24 hours after skin injury in Ly6G^tdTomato^ mice showing neutrophils (red) and blood vessels (cyan). Dotted black lines represent the surrounded neutrophils. (c) Neutrophil numbers in skin wounds of WT mice injured at ZT5 and ZT13, and ATI2341-treated mice, quantified by flow cytometry; n=6-7 mice per group. (d) Whole-mount representative images of neutrophil distribution (left) in and around skin wounds of vehicle- and ATI2341-treated CXCR4^ΔN^ mice injured at ZT5, quantified in bars at right; n=6 mice per group (1-2 wounds per mouse). Solid white lines limit the neutrophil ring and dotted white lines the adjacent area to the wound. (e) Representative images (left) and quantification (right) of annexin V signal around skin wounds from neutropenic (Mcl1^fl/fl^) and control mice; n=4-6 mice per group (2-5 wounds per mouse). (f) Representative confocal images (left) and quantification (right) of TUNEL staining for identification of apoptotic areas around skin wounds in control mice (ZT5), mice injured at night (ZT13) and ATI2341-treated mice; n= 3 mice per group (1-2 wounds per mouse). Data in (c and d) are pooled from two experiments. Data in (e) are pooled from four experiments. Data in (f) are from a single experiment. Data are shown as mean ± SEM. *p<0.05; ns, not significant, as determined by one-way ANOVA (c, f) and unpaired t test (d, e).

**Supplemental** **Material**

Figure S1

**
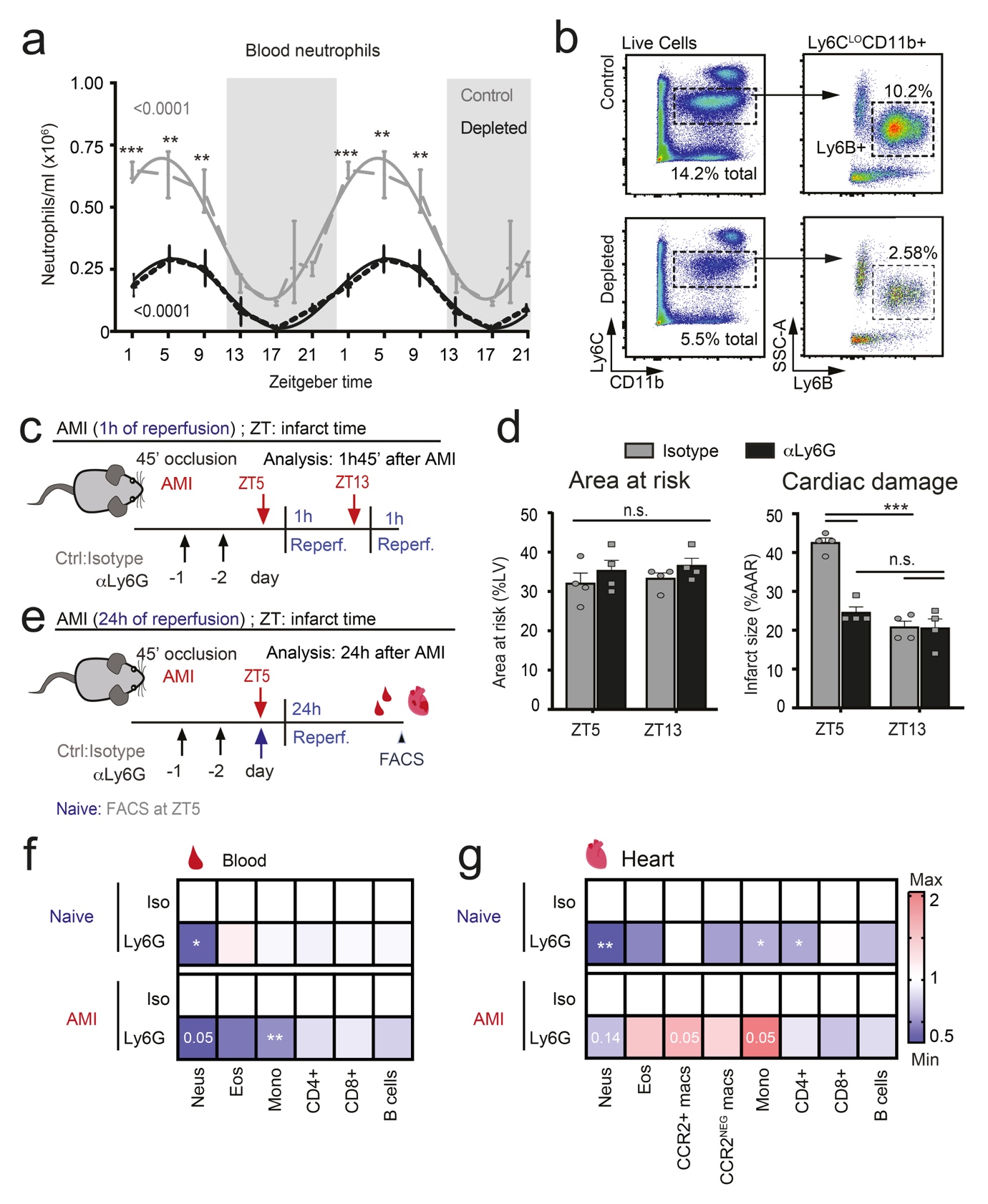
**

Figure S2

**
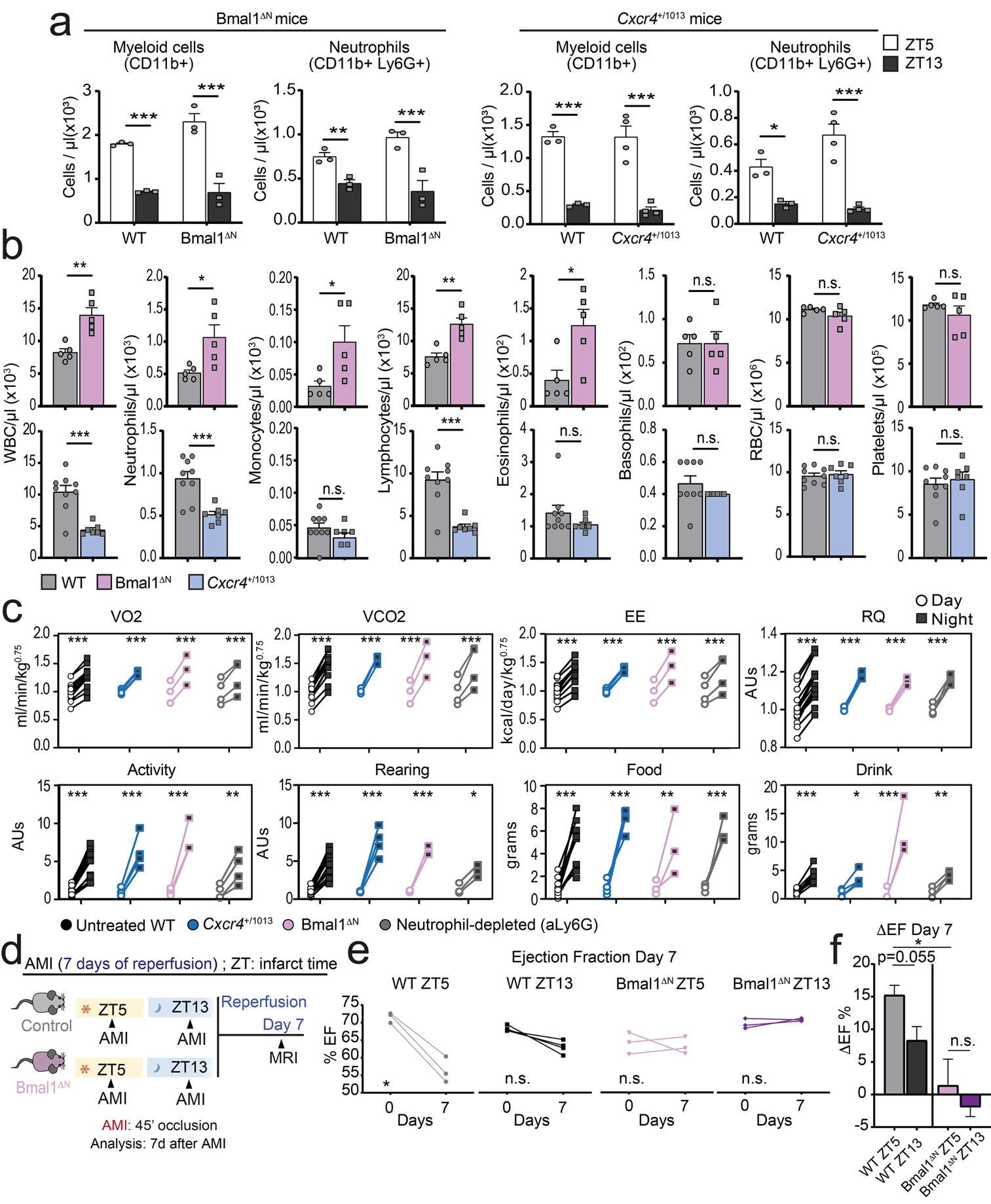
**

Figure S3


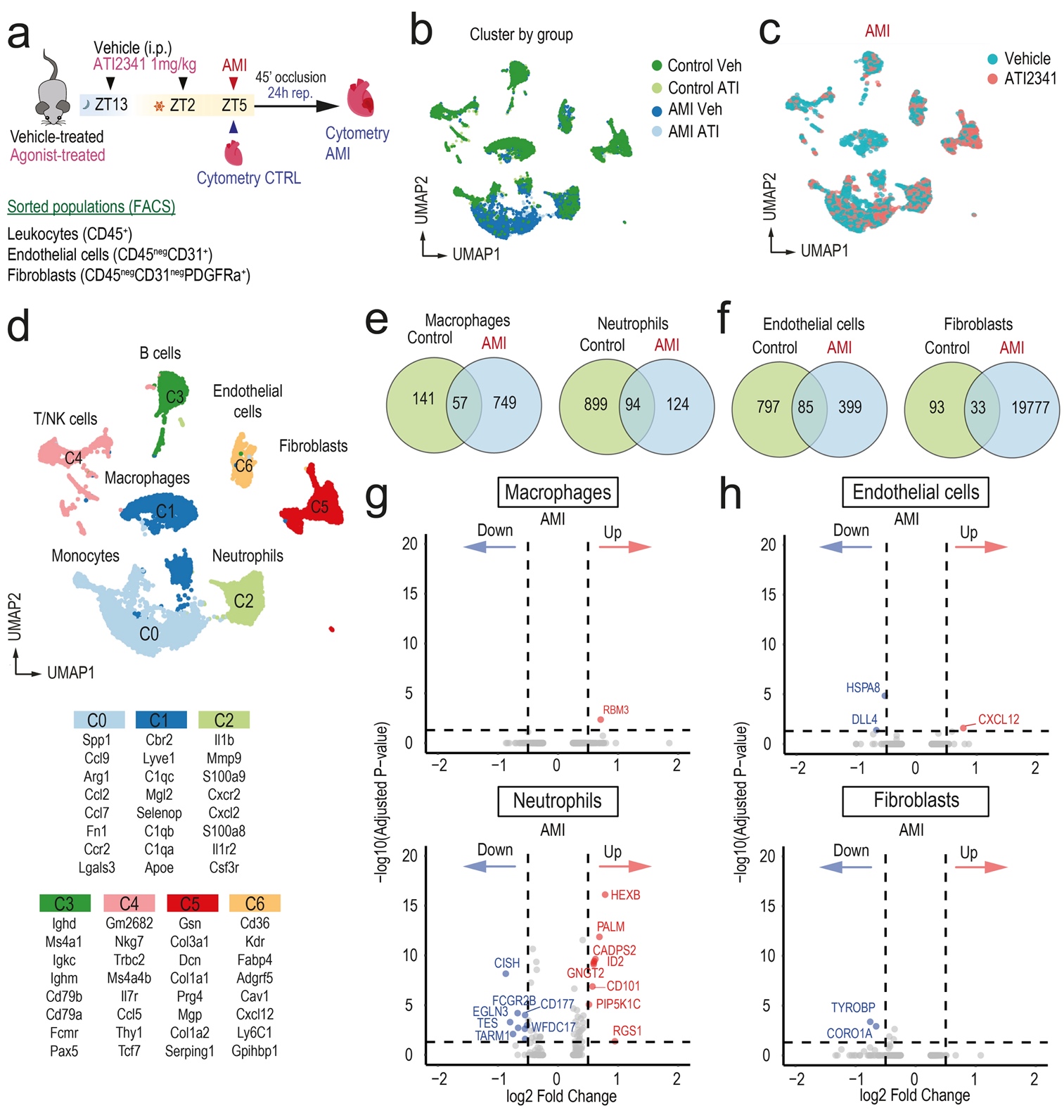


Figure S4


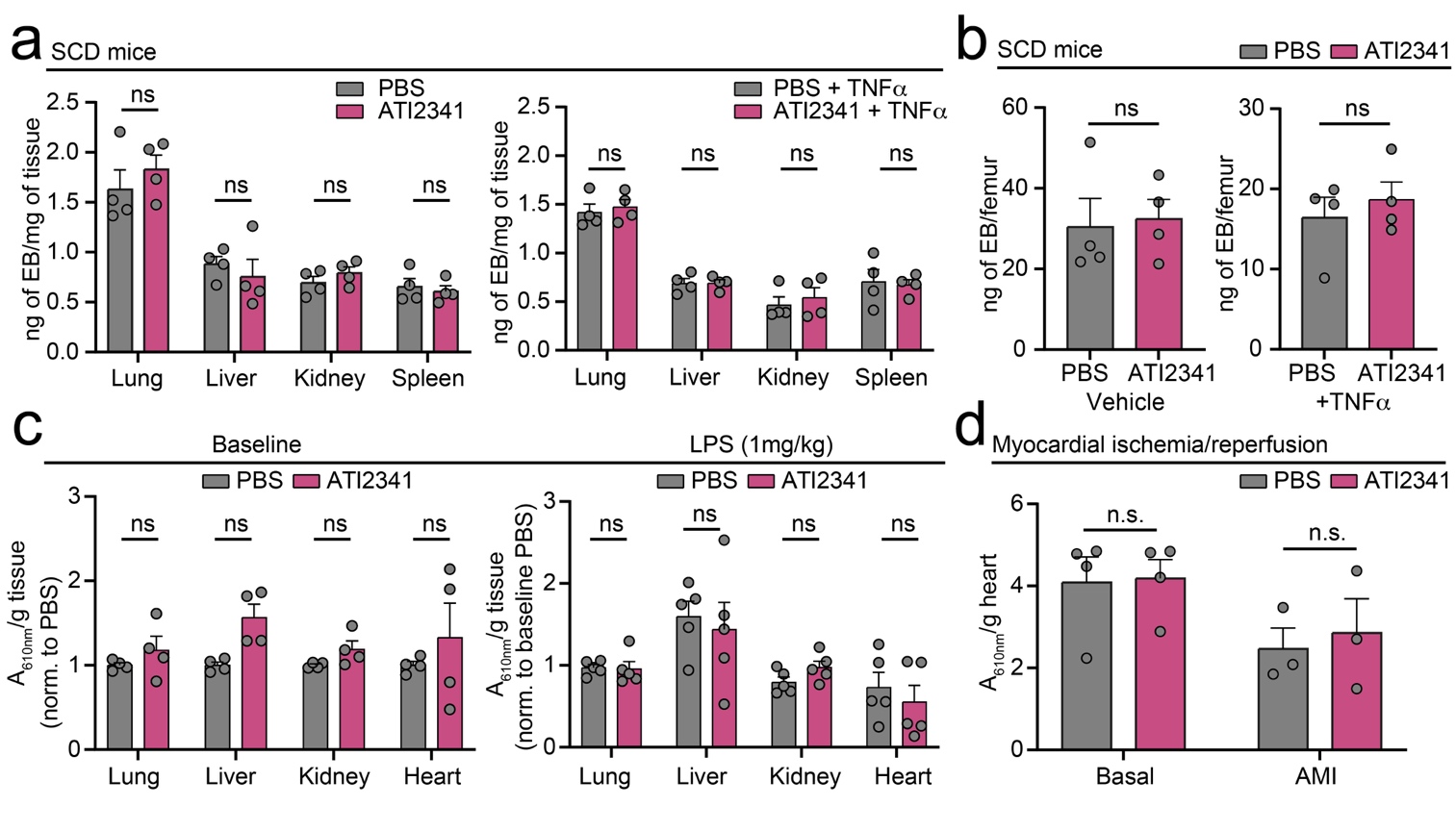


Figure S5

**
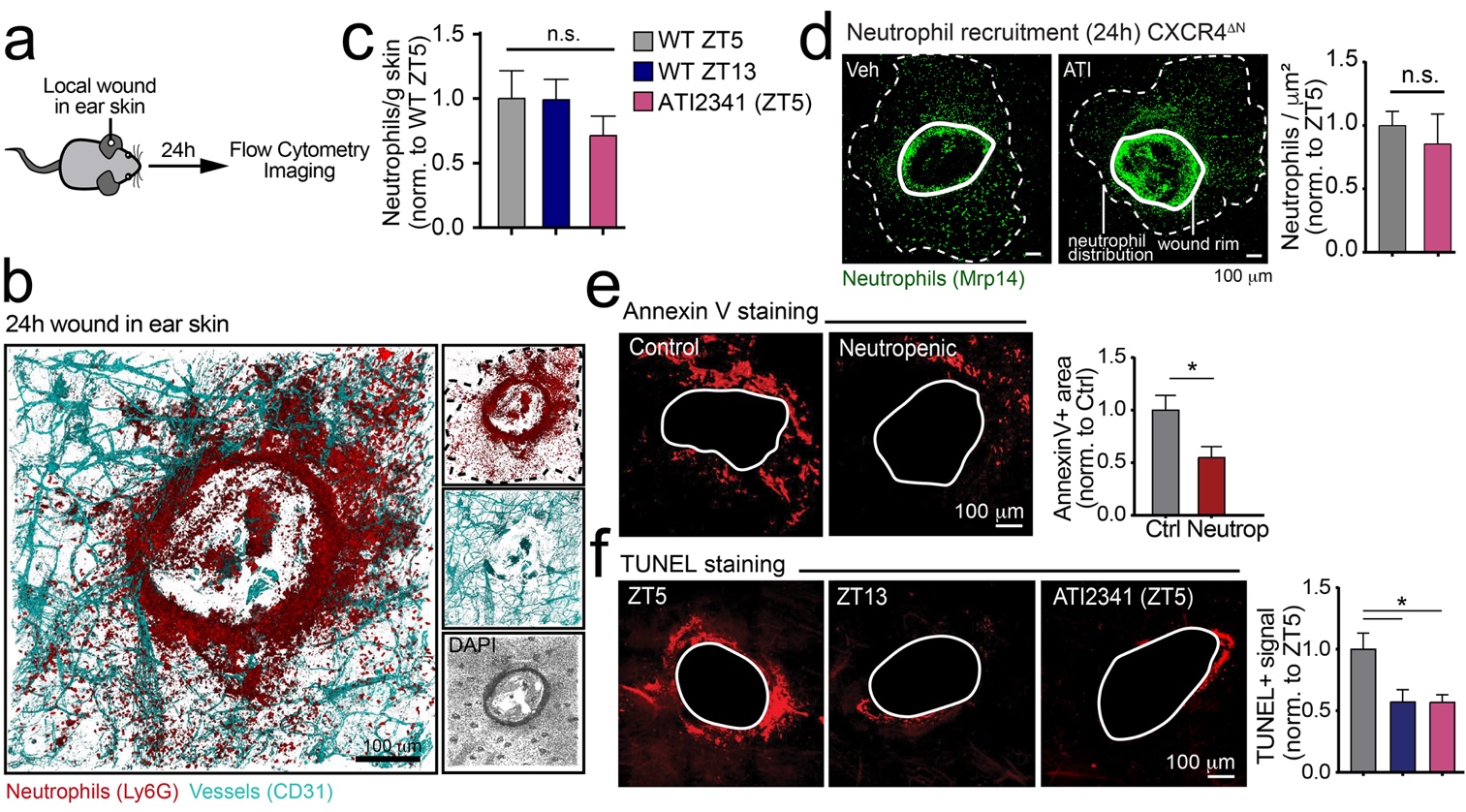
**
